# Whole-genome bisulfite sequencing maps from multiple human tissues reveal novel CpG islands associated with tissue-specific regulation

**DOI:** 10.1093/hmg/ddv449

**Published:** 2015-10-28

**Authors:** Isabel Mendizabal, Soojin V. Yi

**Affiliations:** 1 School of Biology, Georgia Institute of Technology, Atlanta, GA 30332, USA and; 2 Department of Genetics, Physical Anthropology and Animal Physiology, University of the Basque Country UPV/EHU, Barrio Sarriena s/n, 48940 Leioa, Spain

## Abstract

CpG islands (CGIs) are one of the most widely studied regulatory features of the human genome, with critical roles in development and disease. Despite such significance and the original epigenetic definition, currently used CGI sets are typically predicted from DNA sequence characteristics. Although CGIs are deeply implicated in practical analyses of DNA methylation, recent studies have shown that such computational annotations suffer from inaccuracies. Here we used whole-genome bisulfite sequencing from 10 diverse human tissues to identify a comprehensive, experimentally obtained, single-base resolution CGI catalog. In addition to the unparalleled annotation precision, our method is free from potential bias due to arbitrary sequence features or probe affinity differences. In addition to clarifying substantial false positives in the widely used University of California Santa Cruz (UCSC) annotations, our study identifies numerous novel epigenetic loci. In particular, we reveal significant impact of transposable elements on the epigenetic regulatory landscape of the human genome and demonstrate ubiquitous presence of transcription initiation at CGIs, including alternative promoters in gene bodies and non-coding RNAs in intergenic regions. Moreover, coordinated DNA methylation and chromatin modifications mark tissue-specific enhancers at novel CGIs. Enrichment of specific transcription factor binding from ChIP-seq supports mechanistic roles of CGIs on the regulation of tissue-specific transcription. The new CGI catalog provides a comprehensive and integrated list of genomic hotspots of epigenetic regulation.

## Introduction

Since their initial discovery almost three decades ago (1–3), numerous studies have established the critical importance of CpG islands (CGIs) in fundamental regulatory and developmental processes (4–8). Originally defined as hypomethylated stretches of CpG-rich sequences (1–3), CGIs punctuate otherwise heavily methylated, CpG-depleted mammalian genomes (9–13). Cell type- and tissue-specific CGI methylation is a key regulatory signal for genomic imprinting (14), gene expression regulation (4) and developmental programming (5,7,11,15). Aberrant CGI methylation is implicated in numerous diseases, particularly cancers (16,17) and neurodevelopmental disorders (18).

Even though CGIs were originally experimentally defined (1), subsequent annotations of CGIs relied on sequence-based computational algorithms, due to the lack of actual DNA methylation data (2,19–21). These computational algorithms have been extremely valuable for almost two decades. However, whether computationally identified CGIs truly represent hypomethylated CpG clusters has recently been called into question by genome-wide methylation surveys. For example, substantial numbers of computationally defined CGIs are consistently hypermethylated in several tissues (5,22,23) (i.e. false positives). Moreover, many hypomethylated CpG-rich sequences (representing the very definition of CGIs) are missing from the computationally annotated CGI sets (5,24) (i.e. false negatives). Furthermore, a considerable fraction of CGIs has undergone CpG loss during recent evolution, suggesting that they are constitutively methylated and are not bona fide CGIs (25). With the developments of techniques to identify different types of hypomethylated genomic regions (26–28), it is feasible that the term CpG island itself may even be replaced with some other terms in the future. Nevertheless, CGIs are still one of the most widely analyzed genomic elements in epigenetic analyses, and many commercial toolkits preferentially target them (23). Consequently, re-visiting the epigenetic definition of CGIs and providing an experimentally defined CGI catalog that overcomes the limitations of computational predictions will offer a tremendous resource for advancing our knowledge.

**Figure 1.**
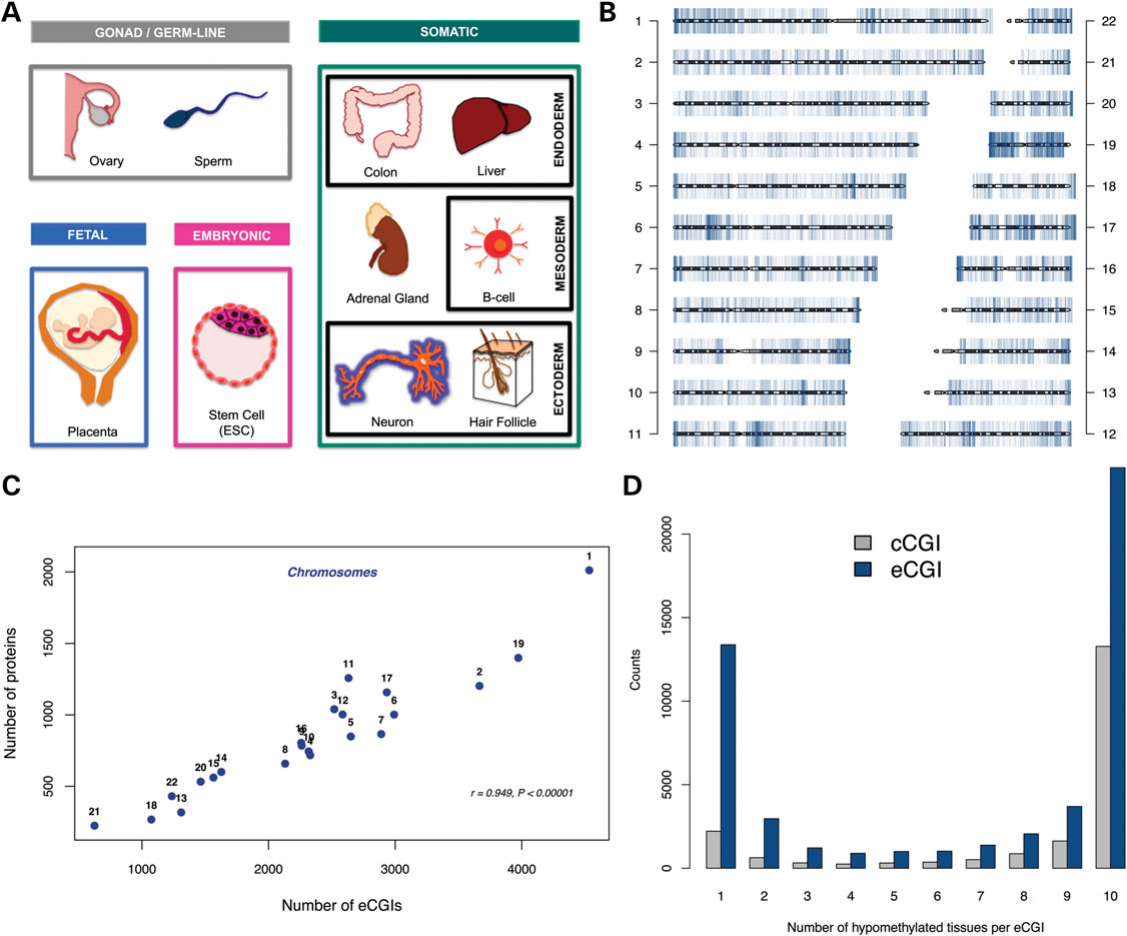
(**A**) Tissues analyzed for eCGI identification, including embryonic, gonad, germ line and fetal tissues, as well as six adult somatic tissues of distinct developmental origins. These were selected to have the highest cell type diversity with respect to gene expression patterns (68) while avoiding overly cell heterogeneous tissues. Ovaries comprise germ-line cells and endoderm-derived tissue. The adrenal gland has both ectodermal (medulla) and mesodermal (cortex) origins. (**B**) The genomic distribution of eCGIs. (**C**) The correlation between the numbers of protein-coding genes and eCGIs on each chromosome. (**D**). Distribution of eCGIs and cCGIs across tissues.

Indeed, important efforts have previously been made to generate an accurate CGI data set (5,22,24). However, these early studies lacked DNA methylation maps with nucleotide-level resolution. They were also limited to only a few tissue types. Here, we utilize whole-genome bisulfite sequencing data sets (11,15,29–34) generated from diverse cell types, including embryonic stem cells (ESCs), germ cells, fetal tissues and six adult somatic tissues spanning all three germ layers (Fig. 1A). From this comprehensive collection of whole-genome methylation maps, we identified more than 50 000 experimentally supported CGIs (‘eCGIs’). The eCGI catalog presented here is the most comprehensive experimentally defined bona fide CGI catalog to date, revealing a large number of novel CGIs that were previously undetected. This experimental definition allows for the discovery of hypomethylated CpG clusters associated with constitutively expressed genes, thereby expanding the list of CGI genes. Moreover, in contrast to the housekeeping nature of classical promoter CGIs, many novel eCGIs show promoter- and enhancer-like chromatin features and associate with facultative transcription factors (TFs) to putatively regulate tissue-specific coding and non-coding transcription.

## Results

### Comprehensive eCGI set

We integrated 10 deep-coverage nucleotide-resolution whole-genome methylation maps to detect eCGIs (Fig. 1A). We used a sliding window approach with 200 bp windows (50 bp step size), with each window containing at least 10 CpGs. We extended the window until it contained <80% of sparsely (<0.2) methylated CpGs. These criteria were selected to provide a fair comparison to computationally identified CGI sets (see Materials and Methods). Results from analyses using other criteria are qualitatively similar and shown in Supplementary Material. Following these procedures, we identified a set of 51 572 non-overlapping eCGIs across tissues (Supplementary Material, Table S1). The genomic distribution of these eCGIs across chromosomes (Fig. 1B) correlates more strongly with gene contents than with chromosome lengths (Pearson's *r* = 0.949, *P* < 10^−5^ and *r* = 0.631, *P* = 0.0016, respectively, Fig. 1C), suggesting that these hypomethylated regions include functional elements. Almost half (46.5%) of the eCGIs are found in all tissues analyzed, whereas over a quarter (25.94%) are tissue-specific (present in only one tissue, Fig. 1D). Consistent with the global hypomethylation of the sperm and placenta genomes (9), most (>80%) of the tissue-specific eCGIs are sperm-specific and another 7.3% are placenta-specific (Supplementary Material, Fig. S1A). In contrast, somatic tissues harbor fewer tissue-specific eCGIs (Supplementary Material, Fig. S1B). Indeed, any somatic sample can recover between 77 and 93% of all somatic CGIs, whereas with four tissues the novel eCGI discovery rate plateaus, indicating that our survey likely identified the majority of somatic eCGIs (Supplementary Material, Fig. S2).

### eCGIs can be used to evaluate and complement computational CGIs

**Figure 2.**
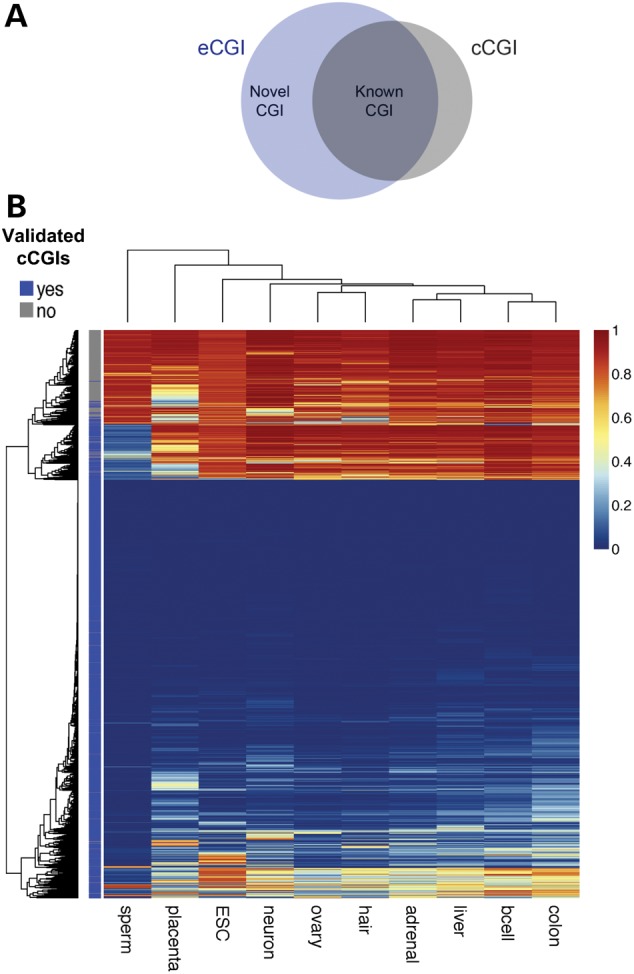
(**A**) Venn diagram showing the overlap between eCGI and cCGI sets. (**B**) Heat map of CpG methylation patterns at cCGIs. cCGIs validated by whole-genome DNA methylation maps are labeled as dark blue, and not-validated cCGIs are shown in gray. Methylation levels are shown as a gradient (blue to red).

**Figure 3.**
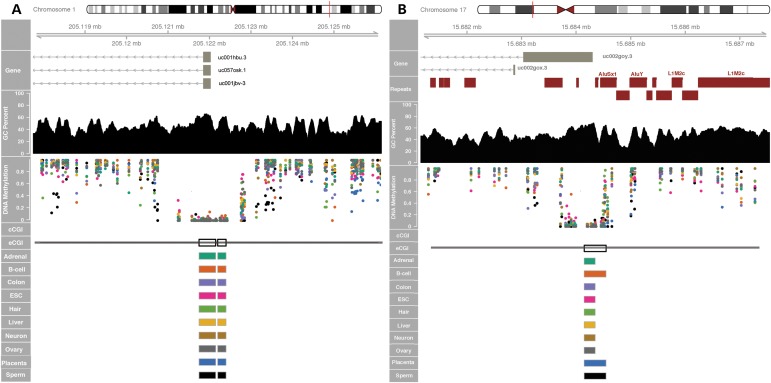
Examples of novel constitutive eCGIs in promoter regions. (**A**) Novel eCGIs are identified in the promoter region of the retinoblastoma binding protein 5 gene *RBBP5* (white blocks) which are not currently annotated in UCSC. Experimental eCGIs are identified using methylation data from individual CpG sites in each tissue (colored dots). (**B**) Novel eCGI at the promoter of the *TRIM16* gene (tripartite motif containing 16) on chromosome 17 overlaps with repetitive elements by repeatmasker (red blocks). These genes are ubiquitously expressed, show active promoter chromatin marks and overlap with TSS in CAGE data in B-cell. Additional examples are shown in Supplementary Material, Figure S4.

One of the most widely used CGI sets is that from the University of California Santa Cruz (UCSC) Genome Browser (25,35,36). These CGIs have been computationally predicted (hereafter referred to as computational CGI, ‘cCGI’) on the basis of the following criteria: a minimum length of 200 bps, a minimum GC content of 50% and an observed/expected ratio of CpG sites above 0.6 (19). We observe an extensive overlap between cCGIs and eCGIs (true positives; 76.1% of the autosomal cCGIs are present in the eCGI set, Fig. 2A). At the same time, however, many cCGIs are not validated by the experimental DNA methylation data (Fig. 2A). Although both sets are identified under the same minimum length criterion, the boundary definition differs. Specifically, the maximum segmental algorithm is used to merge adjacent cCGIs, whereas eCGI boundaries are defined by local methylation values. Consequently, some of these adjacent eCGIs could have arisen due to the lack of coverage in the in-between regions (i.e. Fig. 3A). Nevertheless, the majority of the cCGIs that were not represented in our experimental set showed extensive DNA methylation in all tissues (*n* = 4208, Fig. 2B) and thus are likely false positives. Notably, the current cCGI set fails to identify a substantial number of hypomethylated CGIs (high rate of false negatives), as 39.2% of the eCGIs we report are ‘novel’ (i.e. not on the list of UCSC cCGIs, Fig. 2A). Even if we merge all adjacent eCGIs, of eCGIs remain as novel (Supplementary Material, Fig. S3).

The tissue distributions of cCGIs and eCGIs reveal a striking difference (Fig. 1D). The cCGI sets often occupy eCGIs that are hypomethylated across all tissues examined (referred to as ‘constitutive’ eCGIs), although we also identify many novel constitutive eCGIs that are missed by cCGIs (Fig. 3 and Supplementary Material, Fig. S4). In particular, computational algorithms underperform in the identification of tissue-specific eCGIs. This discrepancy can be at least partially explained by the distinctive DNA sequence characteristics of cCGIs and eCGIs. For example, eCGIs, particularly tissue-specific eCGIs, show significantly lower GC contents and observed/expected CpG ratios (CpG *O/E* ratios) than cCGIs (Supplementary Material, Fig. S5). Approximately 23.45% of the novel eCGIs have GC contents and CpG *O/E* ratios that are below the thresholds used by computational methods, with values as low as 0.36 and 0.40, respectively. Thus, the arbitrary GC content and CpG *O*/*E* ratio thresholds used by computational methods may compromise their power to predict more tissue-specific CGIs.

The presence of repetitive elements is yet another genomic feature that can interfere with the computational prediction of CGIs. Typically, transposable elements are masked for CGI prediction algorithms to avoid false positives (confounding of GC-rich repetitive elements, particularly the Alu family). In contrast, eCGIs substantially overlap with short interspersed repetitive (SINE), long interspersed repetitive (LINE), and long terminal repeat (LTR) elements (Supplementary Material, Fig. S6). For example, we identified a constitutive eCGI in the promoter of *TRIM16*, a widely expressed tumor suppressor-like gene (Fig. 3B). This novel CGI comprises sequences derived from AluYb8 and AluSx1 SINE elements and was thus previously undetected by computational methods that mask repetitive elements. Sperm eCGIs are specifically enriched for SINE-class elements (mostly Alus), as shown previously (33,37,38). Consequently, our experimental approach overcomes the limitations of sequence-based methods to detect CGIs with variable sequence contents and/or repetitive sequences.

### CGI shores and eCGIs

**Figure 4.**
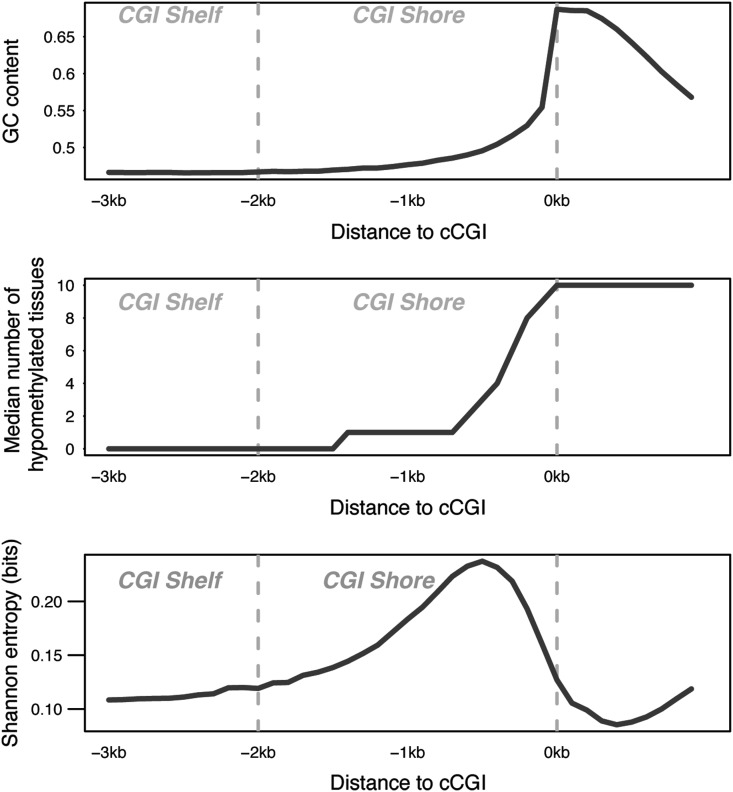
Sequence composition (top), methylation breadth, Shannon entropy (bottom) of cCGIs and upstream CGI shores and shelves.

Early genome-wide methylation studies found that computationally predicted CGIs show relatively low methylation variation across normal and cancerous tissues. In contrast, the regions located immediately upstream (the so-called ‘CGI shores’) were highly variable regarding DNA methylation (39,40). Accordingly, broadly hypomethylated cCGIs tend to be flanked by more tissue-restricted eCGIs located upstream, which concentrate the highest amount of methylation variation (Fig. 4). Interestingly, the GC contents and CpG *O*/*E* ratios in the shores are below the criteria used by computational methods, explaining why computational algorithms typically miss the tissue-restricted islands at ‘CGI shores’ and providing a mechanistic explanation to this phenomenon.

### TF binding potential and chromatin states of eCGIs indicate promoter and enhancer features

**Figure 5.**
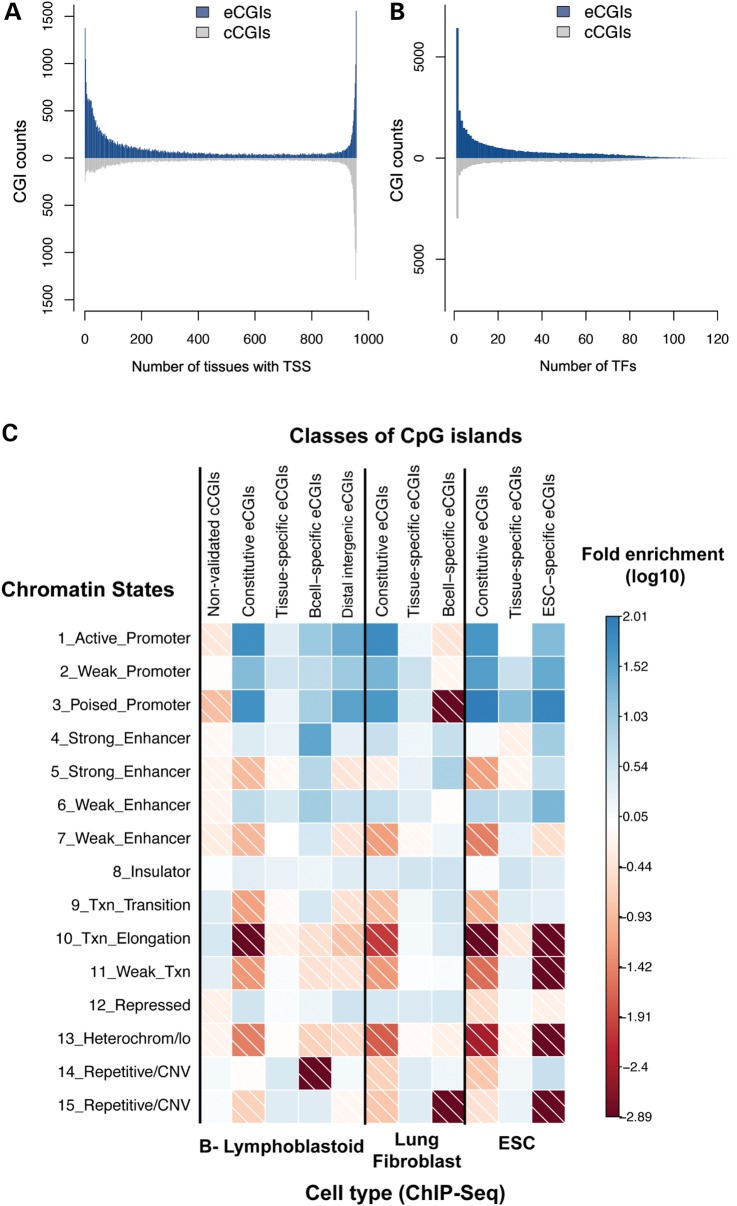
(**A**) Differing counts of TSS in eCGIs and cCGIs. The *X*-axis represents the number of FANTOM5 tissues in which a given CGI harbors one or more TSSs (CAGE data). (**B**) Distinctive TF binding potentials of eCGIs and cCGIs. The *X*-axis represents the numbers of different TFs that bind to each CGI. Data on specific TF binding were obtained from ChIP-Seq data from ENCODE. (**C**) Enrichment for chromatin states in different CGI categories. ChIP-Seq based chromatin state maps for three cell types are shown: B-lymphoblastic cells, lung fibroblasts and ESCs. Colors indicate the log_10_ of the fold enrichment values.

To understand the functional roles of eCGIs, we analyzed several epigenomic and transcriptomic profiles of eCGIs. Experimentally annotated transcription start sites (TSSs) using CAGE data from the FANTOM 5 project (41) show that nearly all eCGIs (98.1%) harbor at least one TSS. Comparison of TSS distributions of cCGIs and eCGIs illustrates different strengths of the two approaches: although cCGIs capture TSSs that are constitutively active in a broad number of tissues, eCGIs excel at identifying tissue-specific TSS (Fig. 5A). Moreover, patterns of DNA methylation at eCGIs are informative of their transcription initiation potential across tissues: the number of tissues where each eCGI is hypomethylated (referred to as ‘hypomethylation breadth’) and tissue-wise distribution of TSS are strongly positively correlated (Spearman's *ρ* = 0.73, *P* < 10^−15^, Supplementary Material, Fig. S7A).

We further examined ChIP-seq data for binding of 161 TFs in 91 cell types (42) to better understand the mechanistic underpinning of the TSS potential of eCGIs. The majority (81%) of eCGIs overlaps with experimentally characterized TF binding regions. Constitutive eCGIs exhibit significantly greater overlap with TF binding regions (99.8%) than tissue-specific ones (37%), as expected given some of our tissues do not have a direct correspondence with ENCODE samples. Tissue distributions of eCGIs show a strong correlation with the number of different TFs they can potentially bind (Spearman's *ρ* = 0.78, *P* < 2.2×10^−16^, Supplementary Material, Fig. S7B). Consequently, constitutively hypomethylated eCGIs appear to be recognized by a wide repertoire of TFs, whereas cell-type specific eCGIs bind to a restricted number of TFs. For instance, B-cell eCGIs are enriched for regions binding key TFs involved in B-lymphopoiesis and lymphoma pathogenesis (such as BCL11A, EBF1, IKZF1 and SPI1), whereas ESC-specific eCGIs are enriched for binding of NANOG, a key TF regulating pluripotency (43,44). Constitutive eCGIs, in contrast, are associated with general TFs such as CREB1 and TAF7 (Supplementary Material, Table S2). TF binding patterns of eCGIs and cCGIs illustrate the enhanced ability of eCGIs to capture genomic loci of higher TF specificity (Fig. 5B).

We also examined chromatin state maps (45) in three cell types: two cell types overlapping with eCGI discovery tissues (B-lymphoblastoid cell and ESC) and a control cell type (lung fibroblast). Constitutive eCGIs are associated with promoter-related chromatin states (regions with high frequencies of H3K4me2 and H3K4me3 marks) in all three tissues (e.g. >55-fold in B-cells and >65-fold in lung fibroblasts, Fig. 5C). In contrast, tissue-specific eCGIs are over-represented by enhancer marks, but only at concordant tissues. For example, B-cell-specific eCGIs are highly enriched for strong enhancer chromatin marks in B-lymphoblastoid cells (>32-fold, *P* < 0.001, high frequencies of H3K4me1, H3K4me2, H3K4me3, H3K27ac and H3K9ac), but not at lung fibroblasts (4-fold decrease, Fig. 5C). Accordingly, upon studying all tissue-specific eCGIs (including tissue-specific eCGIs in other cell types), the enhancer association is significantly diluted (Fig. 5C). These are consistent with the high cell type specificity of enhancer marks (45). In contrast, cCGIs that are not validated in our experimental data set (putative false positives) do not show enrichment for promoter- or enhancer-like chromatin marks (e.g. strong enhancer: 0.77-fold enrichment, *P* = 0.9). Instead, these cCGIs are slightly associated with transcription-related states (transcription elongation: >3- and >6-fold in B-lymphoblastoid cells and lung fibroblasts, respectively, *P* < 0.001), which can be explained by coding cCGIs’ proclivity for false positives (discussed subsequently).

### Distribution of eCGIs to annotated genomic regions

**Figure 6.**
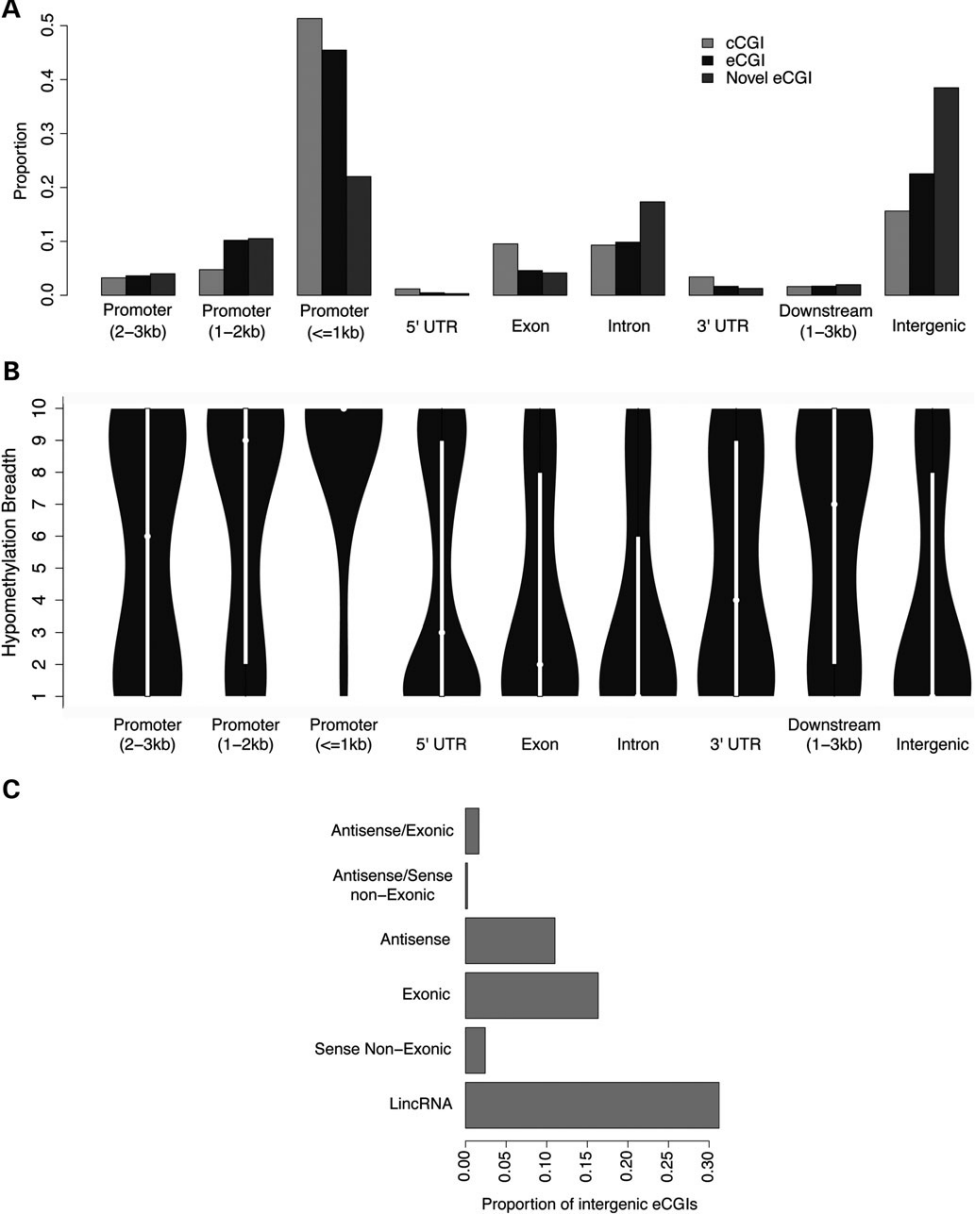
(**A**) Distribution of CGIs in relation to the UCSC annotation of genes. The promoter region was defined as 3 kb upstream of the TSS, downstream region as immediate downstream genes within 3 kb and intergenic regions for distances >3 kb from genes in both directions. (**B**) Hypomethylation breadths (number of tissues with eCGIs) of different regions. (**C**) Proportion of ncRNA classes found within 3 kb of intergenic eCGIs.

When annotated according to the TSSs of transcripts in the UCSC database, the majority of eCGIs are located within genes or within 3 kb of TSSs (63.6 or 59.3%, respectively), consistent with the classical notion of an association between CGIs and gene promoters (Fig. 6A). Gene expression and gene promoter methylation are significantly negatively correlated, represented as a clear ‘L’ shape curve for all tissues (Supplementary Material, Fig. S8), in line with previous studies (46). Heavy promoter methylation is clearly repressive, whereas weak promoter methylation is associated with highly variable gene expression patterns. As expected, genes that are differentially methylated between tissues also tend to be differentially expressed in the expected negative direction across tissue pairs in both microarray and RNAseq data sets (Supplementary Material, Table S3).

It was previously shown that genes harboring cCGIs in promoter regions are more broadly expressed than those without cCGIs (8,47,48). Consequently, promoter CpG content was considered as a proxy for gene expression breadths (47–49). Given that the underlying idea for the association between promoter CpG content and gene expression breadth is DNA methylation, we hypothesized that DNA methylation characteristics of eCGIs in promoters may provide a better indicator of gene expression breadths than CpG content. Indeed, the correlation between DNA methylation breadth and gene expression breadth was about twice as stronger as that between promoter CpG contents and gene expression breadths (Supplementary Material, Table S4). Furthermore, genes with tissue-specific eCGIs in their promoters exhibit more tissue-specific expression than those with constitutive eCGIs (Supplementary Material, Fig. S9) and associate with tissue-specific GO categories (Supplementary Material, Table S5), indicating co-regulation between eCGI methylation and gene expression breadths.

A substantial number of eCGIs (*n* = 8527) are found in gene bodies, where the proportion of novel eCGIs is high (Fig. 6A). These eCGIs tend to exhibit higher tissue specificity of DNA methylation than those at other locations (Fig. 6B). Together with the pervasive presence of TSSs at eCGIs, this finding is consistent with the putative role of gene body eCGIs in the transcriptional initiation of alternative transcripts in a more tissue-specific manner than eCGIs in canonical promoters (50,51). For example, the well-known alternative promoters in the autism-associated gene SHANK3 (50) are annotated as eCGIs. In contrast, most exonic and intronic cCGIs could not be experimentally validated (58.6 and 56.7%, respectively, Supplementary Material, Fig. S10). Computational algorithms appear to particularly underperform within gene bodies, possibly because coding sequences generally have higher GC and CpG contents than the genomic background. High false positive cCGIs in exons and introns also explain why they exhibit transcription-related chromatin features (Fig. 5C).

The functional role of intergenic eCGIs is of particular interest, as almost 40% of the novel eCGIs are located ≥3 kb from the closest annotated gene, far outnumbering the currently annotated cCGIs in intergenic regions (4975 predicted versus 12 816 experimentally validated, Fig. 6A). The hypomethylation breadth of intergenic eCGIs decreases with the distance to the nearest gene (Supplementary Material, Fig. S11), indicating that eCGI hypomethylation is more tissue-specific in gene deserts. Chromatin states of distal intergenic eCGIs (>10 kb from any gene, *n* = 9353) associate with promoter and enhancer features (Fig. 5C). Notably, even novel distal intergenic eCGIs associate with promoter- and enhancer/insulator-related chromatin states (∼48 and ∼13% of eCGIs, respectively, active promoter: >28-fold enrichment, *P* < 0.001 in B-lymphoblastoid cells, Supplementary Material, Table S6). These features of intergenic eCGIs indicate their potential to enhance or initiate transcription in a more tissue-specific fashion than promoter eCGIs. Indeed, we find that 27.5% of the intergenic eCGIs overlap with non-coding RNAs (ncRNAs) in the NONCODE V4 database (52) and 43.1% of the intergenic eCGIs have an ncRNA within 3 kb. Among these, the most common type of ncRNA is long intergenic ncRNA (lincRNA) (Fig. 6C). Considering the global presence of TSS at eCGIs and the fact that non-coding transcripts are generally more tissue-specific than coding genes [78 versus 19% in the case of lincRNAs (53)], additional associations between eCGIs and ncRNAs are likely to be identified as more tissues are included in the eCGI discovery. These features of intergenic eCGIs are consistent with the role of ncRNAs in transcriptional regulation (54,55).

### Some eCGIs exhibit patterns consistent with genomic imprinting

**Figure 7.**
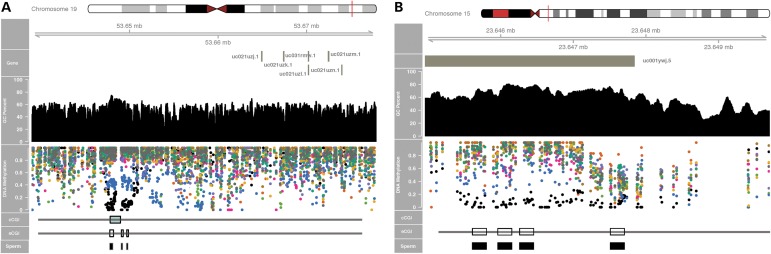
(**A**) Imprinted CGI in the upstream of C19MC miRNA cluster is annotated as eCGIs. (**B**) Cluster of four novel eCGIs in the imprinted gene MAGEL2. The color scheme for DNA methylation values (dots) is identical to that in Figure 3.

Comparison of DNA methylation patterns of sperm versus other tissues is consistent with genomic imprinting at some eCGIs. For example, the human miRNA cluster C19MC comprises dozens of primate-specific ncRNAs (56) that are silenced in normal adult tissues, but are expressed in the placenta, sperm and in many tumor cells (56,57). The eCGIs upstream of C19MC (Fig. 7A) exhibit sperm-specific hypomethylation (black dots) and a hemi-methylated pattern unique to the placenta (blue dots). These are consistent with the maternal imprinting of this region in the placenta (57). Thus, the eCGI catalog may include yet unidentified imprinted CGIs. For instance, we find several novel eCGIs in the MAGEL2 gene, which associates with the Prader–Willi syndrome and shows a paternal-specific expression in placenta (58), in line with the methylation patterns of these eCGIs (Fig. 7B). These examples illustrate that germ-line-specific eCGIs could be useful to localize novel candidates for imprinting and/or to identify tissues at which parent-of-origin expression might occur.

## Discussion

Variation of DNA methylation patterns at CGIs is critical in many aspects of biological processes, particularly in development and disease (1,3,17,21). Even though originally proposed as ‘epigenetic’ regulatory marks of the genome (1), most widely used list of CGIs is predicted from computational algorithms. Such cCGIs have been widely employed in epigenetic studies during the last two decades. However, recent analyses indicate that cCGIs harbor very low epigenetic variability, with only a small portion of CGIs presenting tissue-specific hypomethylation (5,23,59). Here, we demonstrate that an experimental approach can be adopted to successfully account for the variation in DNA methylation at CGIs and to overcome the limitations of bioinformatic methods.

We identified numerous hypomethylated CGIs that are experimentally validated (eCGIs). Many of these eCGIs are not included in the current CGI annotation in UCSC. Further analyses support the idea that the arbitrary GC content and CpG *O*/*E* ratio thresholds used by computational methods compromise their power to predict more tissue-specific CGIs (5,22,23). In contrast, computational algorithms tend to misidentify coding sequences with high GC and CpG contents as CGIs, even though they are constitutively hypermethylated (false positives).

In addition, we find significant impacts of transposable elements on the distribution of eCGIs. Recent reports suggest tissue-specific enhancer activity of hypomethylated transposable elements (60). However, repetitive sequences are typically masked for cCGI prediction to avoid false positives (21). The eCGIs from whole-genome bisulfite sequencing data affirm a pervasive presence of repetitive elements on eCGIs, revealing a large number of repeat-derived hypomethylated regions. Many of them harbor enhancer and/or promoter features, highlighting the significant and dynamic role of repetitive elements in constructing the epigenetic landscapes of the human genome (61,62).

Comparative analyses of eCGIs and cCGIs provide insights into their complementary functional nature. eCGIs that are hypomethylated in all samples, or ‘constitutive’ eCGIs, generally overlap with cCGIs. In contrast, many novel eCGIs exhibit highly tissue-specific patterns of hypomethylation and show little overlap with cCGIs. Analyses of TSS and TF binding potentials substantiate the link between hypomethylation and the initiation of transcription. Nearly, all eCGIs harbor experimentally annotated TSSs, and their tissue-wise hypomethylation patterns mirror the patterns of TSS distribution. For instance, many novel eCGIs harbor TSSs that are active in a limited number of tissues, consistent with the idea that they are involved in tissue-specific transcription. Moreover, tissue-specific eCGIs are enriched with regions binding highly specialized TFs, whereas constitutive eCGIs bind to a slew of general TFs. These characteristics, and comparisons to cCGIs, demonstrate that eCGIs excel at identifying loci associated with tissue-specific regulation of transcription.

Thus, whole-genome methylation maps provide a comprehensive and experimentally validated list of hypomethylated CGIs that are complementary to the widely used cCGI sets. In particular, we substantially update the list of CGIs within gene bodies, in which computational algorithms particularly underperform due to the confounding effects of sequence composition. We also list numerous hypomethylated CGIs in intergenic regions, which often harbor chromatin marks that are consistent with promoter and enhancer features. An eCGI catalog ascertained from a wide range of cell types including germ lines can additionally shed light on the genomic regions involved in allele-specific epigenetic processes such as genomic imprinting.

Previous efforts to define CGIs using experimental methods relied on CXXC binding (22,24). Genome-wide differences in chromatography affinity and CpG density, however, might affect the efficiency of CGI discoveries (63). Indeed, we observe distinctive sequence features at these previous eCGI catalogs (Supplementary Material, Table S7 and Fig. S12). In contrast to affinity-based methods, whole-genome bisulfite sequencing technique provides DNA methylation values of nearly all CpG dinucleotides, independently of its methylation status and (largely) of sequence content, allowing an unbiased and reproducible CGI identification. eCGIs also provide improved annotations of functional elements compared with previous methods (Supplementary Material, Table S8). For instance, promoter and enhancer/insulator chromatin marks occupy 68 and 39% of the eCGI lengths, respectively, highlighting the benefits of nucleotide-resolution maps for delimiting hotspots of epigenetic regulation.

One caveat of our analyses is that we included tissues from different individuals that might harbor single nucleotide polymorphisms (SNPs) (C to T mutations), compared with the reference genome. Because CpG sites are particularly prone to point mutations (64,65), SNPs can affect the inference of DNA methylation. Ideally, to circumvent this issue, genomic and epigenetic profiles from same individuals should be used (27) or in case of deeper sequencing coverage data, use mapping tools that will take into account SNPs and DNA methylation in parallel (66). Given the uncertainty about the samples and due to limited coverage, we discarded sites known to harbor such SNPs based on the 1000 Genomes Project data, to partially circumvent this problem (26,67). Nevertheless, the resulting eCGI set exhibits signatures of functional and regulatory elements from several independent data sets, indicating that our strategy successfully identified epigenetically consistent profiles.

In summary, we describe a novel, extensive catalog of eCGIs that curates the currently used CGI sets and adds the critical tissue dimensionality that is inherent to any epigenetic study. This CGI catalog maintains a total length of 20.9 MB, which is equivalent to that of the currently annotated CGIs in the UCSC Genome Browser. Our comparison to USCS CGIs might be conservative because other prediction algorithms have higher false-positive rates (2). Being based on bisulfite sequencing (the gold standard technique for the study of DNA methylation), the present CGI set provides the highest resolution, improving the annotation of regulatory elements within CGIs. We expect this CGI catalog to be a valuable resource for epigenetic studies and therefore recommend its use in DNA methylation-reduced representation assays and methylation arrays targeting tissue-specific regulation.

## Materials and Methods

### Bisulfite sequencing data

We downloaded whole-genome bisulfite sequencing reads for 10 different tissues (Supplementary Material, Table S9). The tissues were selected to have the highest cell type diversity with regard to global gene expression (68), and we avoided heterogeneous cell types and *in vitro* cultured cells whenever possible (the ESC sample is the only cultured sample included). The selected tissues also included different early embryonic primary cell layers (endoderm, mesoderm and ectoderm). We followed the quality control steps recommended for bisulfite sequencing data (69) to ascertain the accuracy of the methylation calls. We applied quality control and mapping procedures to all samples to obtain a homogeneous, high-quality data set (Supplementary Material, Table S10). The reads were aligned to the Human Reference Genome (hg38) using Bismark (70). We considered 18 889 743 CpG sites that were covered by at least five reads in all 10 tissues. The median read depth coverage of these sites was >12× (Supplementary Material, Table S11). Fractional methylation levels were computed as the ratio of the counts of methyl-C to the total counts of C for the genomic region of interest (12,13,15).

### CGI detection

To avoid the confounding effects of DNA methylation and sex chromosomes (13), only autosomes were considered for CGI detection. Given the uncertainty about the samples and due to limited coverage, we discarded polymorphic CpG sites overlapping with SNPs at >1% minor allele frequency in the 1000 Genomes Project data (71). To avoid genomic regions that cannot be unambiguously mapped, CpGs with Genome Mappability Scores below 50 were also discarded (72). A total of 18 009 699 CpG sites remained.

We identified eCGIs in each of the 10 methylomes using a sliding window approach with in-house PERL scripts. We used a 200 bp sliding window with a 50 bp step size and extended the window until it contained <80% of sparse (<0.2) methylation. The windows also had to include at least 10 CpG sites (we also considered other densities, see CpG density and CGI definition). The length criterion of 200 bp was chosen for a fair comparison to the cCGIs in UCSC. Under these conditions, the CpG *O*/*E*s of each window ranged between 0.2 and 20. Following these procedures, we detected approximately 30 000 eCGIs per tissue, ranging between 200 bps and 3.6 kbps in length (Supplementary Material, Fig. S13). To generate the final eCGI set, the eCGIs that overlapped across tissues were merged (345 475 CGIs in 10 tissues were merged to 51 572 CGIs).

### CpG density and CGI definition

We explored the impact of different criteria on eCGI discovery. Changing the lengths and/or the hypomethylation criteria did not substantially change the number or location of the CGIs (Supplementary Material, Table S12). The most significant factor on the eCGI sets is the CpG density. As expected, a low CpG density allowed for the inclusion of a higher number of hypomethylated islands, ranging from almost 500 000 eCGIs (spanning 173 MB), in contrast to the conservative set of 29 000 CGIs (9.7 MB, Supplementary Material, Table S13) on the other extreme. The setting of at least 10 CpGs per window led to a total of 20.89 MB of genomic eCGIs, which is comparable to the UCSC cCGI set (20.92 MB), allowing for a fair comparison for the purposes of this study. Lower CpG density allows the discovery of additional tissue-specific eCGIs (Supplementary Material, Table S13).

### CGI genomic features

Statistical analyses were performed using R (73). Repetitive elements were downloaded from UCSC (rmsk table hg38). Overlaps among the tissue-level eCGIs with cCGIs as well as with other genomic features were computed using the GenomicRanges (74) R Bioconductor package. All genomic coordinates from previous builds were converted to hg38 using the UCSC liftover tool in rtracklayer R package. Venn diagrams were computed using the venneuler package. The distribution of CGIs within 3 kb of the TSS of the closest gene (the longest transcript of each gene) was computed with the ChIPseeker package and using the KnowGene table in UCSC hg38. Intergenic eCGIs are defined as those that are at least 3 kb away from any gene region. GO category enrichment analyses were performed with the GOstats package (75). For plotting, we used the Colorbrewer, Gviz and corrplot packages. Shannon information was computed as −*P* × log_2_(*P*), in which *P* is the ratio of the number of tissues showing hypomethylation (<0.2) to the total number of tissues studied.

### Gene expression and transcription initiation

Gene expression data were downloaded from the BioGpS database (http://biogps.org/). The data set comprised GC Robust Multi-array Average normalized Affymetrix microarray data from 78 healthy tissues (HumanU133A/GNF1H Gene Atlas). The 44 775 probes were assigned to 12 494 Entrez genes using the Bioconductor Affymetrix Human Genome U133A 2.0 Array annotation library. The expression breadth of each gene was computed as the number of tissues showing expression values above the median across all tissues and genes. Using different cutoffs (0.25 and 0.75 quantiles) provided similar results (Supplementary Material, Fig. S9). RNAseq data sets were downloaded from the RNAseq Atlas (http://medicalgenomics.org/rna_seq_atlas), and the following tissues were included: colon, liver, ovary and hypothalamus (matched with a neuron sample for methylation). CAGE data from FANTOM5 (41) were downloaded using the CAGE Bioconductor package. Different cutoffs for the number of minimum tags supporting each TSS (from 1 to 5) showed similar distribution of TSS breadths (Supplementary Material, Fig. S7C and D).

### TFs and chromatin states

We analyzed ENCODE ChIP-Seq data for 161 TFs in 91 cell types (wgEncodeRegTfbsClusteredV3 table in UCSC). We used the chromatin states for B-lymphoblastoid cells (GM12878), ESCs (H1 ES) and lung fibroblasts (NHLF) (wgEncodeBroadHmm table in UCSC). These chromatin states were inferred from high-throughput sequencing (ChIP-Seq) experiments on the following chromatin marks: H3K4me3, H3K4me2, H3K4me1, H3K9ac, H3K27ac, H3K36me3, H4K20me1, H3K27me3 and CTCF (45). The enrichment and *P*-values were obtained by bootstrapping. For each CGI category, the same number and width of islands were randomly sampled 1000 times from the genome-wide chromatin states.

### ncRNA databases

We downloaded the NONCODE V4 data set from http://www.noncode.org (29 October 2015, date last accessed). To test the significance of the overlap with intergenic eCGIs, we applied the *χ*^2^ test, considering only autosomal chromosomes and assuming 0.89 as the mappable fraction of the genome.

### Data availability

The annotations (hg38) for the experimentally defined CGIs (eCGIs) and their tissue-wise distribution (1: presence, 0: absence) are shown in Supplementary Material, Table S1.

## Supplementary Material

Supplementary Material is available at *HMG* online.

## Funding

This work was supported by a post-doctoral grant from the Basque Government (Research Personnel Improvement Program by the Department of Education, Language Policy and Culture) (POS_2013_1_130, POS_2014_2_49 to I.M.) and Georgia Tech-Atlanta Zoo collaborative grant, National Science Foundation (BCS-1317195 to S.V.Y.) and the National Institutes of Health (R21MH102677 and 1R01MH103517-01A1 to S.V.Y.). Funding to pay the Open Access publication charges for this article was provided by the National Institute of Health grant 1R01MH102677-01A1.

## Supplementary Material

Supplementary Data
