# Supplementary material for: Whole-genome bisulfite sequencing maps from multiple human tissues reveal novel CpG islands associated with tissue-specific regulation
Source: Hum Mol Genet. 2015 Oct 28;25(1):69–82. doi: 10.1093/hmg/ddv449 (PMC4690492; doi:10.1093/hmg/ddv449)
Supplement: Supplementary Data [file supp_25_1_69__index.html]

Whole-genome bisulfite sequencing maps from multiple human tissues reveal novel CpG islands associated with tissue-specific regulation — Whole-genome bisulfite sequencing maps from multiple human tissues reveal novel CpG islands associated with tissue-specific regulation — Whole-genome bisulfite sequencing maps from multiple human tissues reveal novel CpG islands associated with tissue-specific regulation — Supplementary Data 

# Whole-genome bisulfite sequencing maps from multiple human tissues reveal novel CpG islands associated with tissue-specific regulation

## Supplementary Data

Supplementary Data

- Supplementary Data - Pdf file
- Supplementary Table 1 - txt file
