# Supplementary material for: Whole-genome bisulfite sequencing maps from multiple human tissues reveal novel CpG islands associated with tissue-specific regulation
Source: Hum Mol Genet. 2015 Oct 28;25(1):69–82. doi: 10.1093/hmg/ddv449 (PMC4690492; doi:10.1093/hmg/ddv449)
Supplement: Supplementary Data [file supp_ddv449_ddv449supp.pdf]

SUPPLEMENTARY DATA

PAGES

1-2 **INDEX**

3-19 **SUPPLEMENTARY FIGURES**

Figure S1. Tissue distribution of eCGIs.

Figure S2. CGI discovery rate with increasing number of tissues.

Figure S3. Autocorrelation values of CpG methylation values in chr 19 in Bcell.

Figure S4. Novel constitutive eCGIs in promoters of genes.

Figure S5. Sequence contents of computational (cCGI) and experimental CGI (eCGI) sets.

Figure S6. Repetitive elements (repeatmasker) at CGIs.

Figure S7. Hypomethylation breadth and number of TSS and TFs they harbor

Figure S8. Correlation between promoter methylation and gene expression.

Figure S9. Expression breadth of tissue-specific eCGIs versus constitutive eCGIs.

Figure S10. Fraction of non-validated (methylated) computational CGIs according to different genomic regions.

Figure S11. Number of tissues that show eCGI hypomethylation according to distance from nearest gene.

Figure S12. Comparison of experimental CGI sets.

Figure S13. eCGI characteristics per tissue.

Figure S14. Tissue distribution of eCGIs defined by using different CpG density criteria.

20-32 **SUPPLEMENTARY TABLES**

TableS1.txt **Table S1.** Annotations for the experimentally defined CpG islands (eCGIs) and their tissue-wise distribution (1: presence, 0: absence).

Table S2. Enrichment for transcription factors at different CGI classes.

Table S3. Correlation values (Spearman's rho) between the differential

promoter methylation and gene expression of tissue pairs.

Table S4. Correlation between sequence content of promoter regions and methylation breadth with gene expression breadth

Table S5. GO enrichment (Biological Process) for CGI promoter genes.

Table S6 Enrichment values of different chromatin states at novel intergenic eCGIs

Table S7. Comparative table for studies of experimentally defined CGIs.

Table S8. Functional fraction of CGIs from different catalogues.

Table S9. Samples used in this study.

Table S10. Number and length of reads analyzed per tissue at each step of QC and mapping.

Table S11. Statistics on mapping and methylation calling.

Table S12. eCGI discovery according to different settings.

Table S13. eCGI discovery and functional overlap according to different settings of CpG density.

Supplementary Figures

Figure S1. A. Histogram showing the tissue distribution of tissue-specific CGIs. B. Venn diagram showing the sharing of eCGI across different tissue types. Somatic tissues include the following 7 tissues: adrenal, b-cell, hair follicle, liver, neuron, and ovary.

A

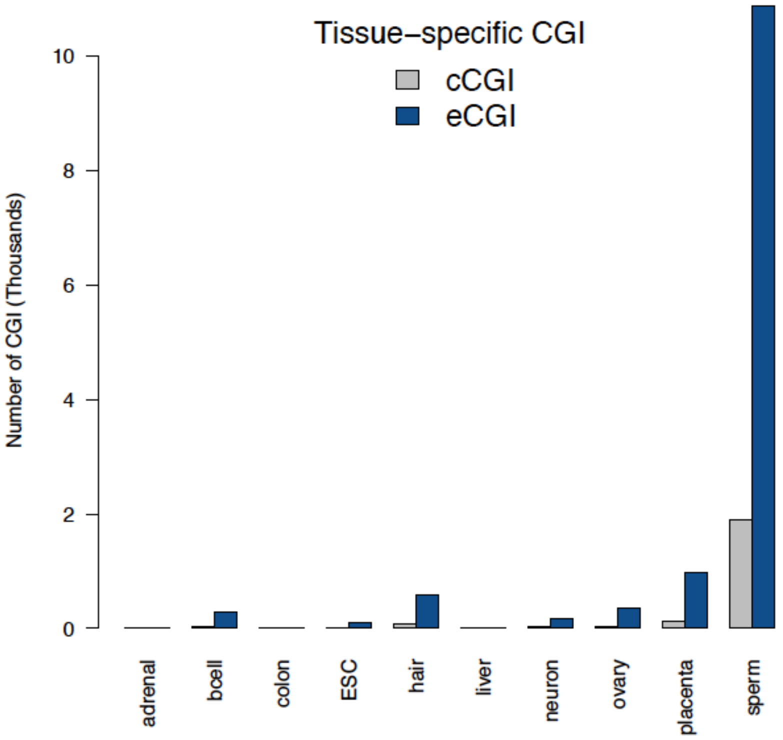

B

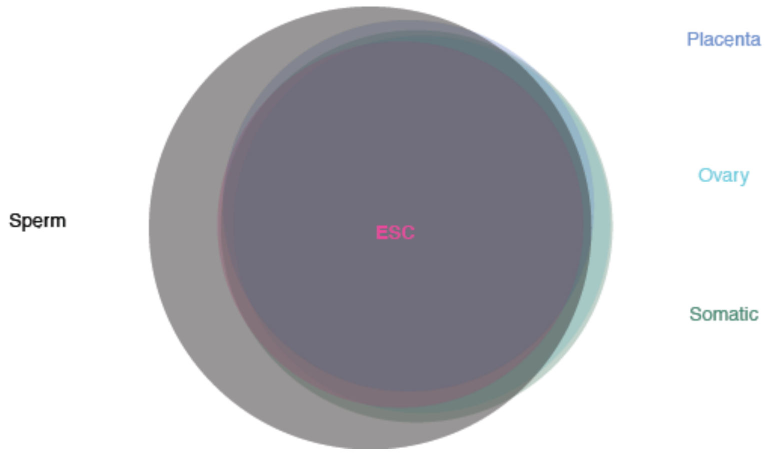

Figure S2. Figure showing the percentage of CGI discovered (y axis) as the number of tissues studied increases (x axis). We randomly took two of our tissues and checked the percentage of novel eCGIs that are discovered progressively as more tissues are added. For the black dots, the 100% in the Y axis represents the total eCGIs identified only using somatic tissues. For white dots, the 100% of total number of eCGIs also includes ESC, placenta, ovary and sperm eCGIs. The figure shows that over 85% of somatic eCGIs are discovered only using 2 tissues (black line). In contrast, sperm-specific eCGIs (not present in other tissues) enclose above 21% of total eCGIs identified in this study (white line).

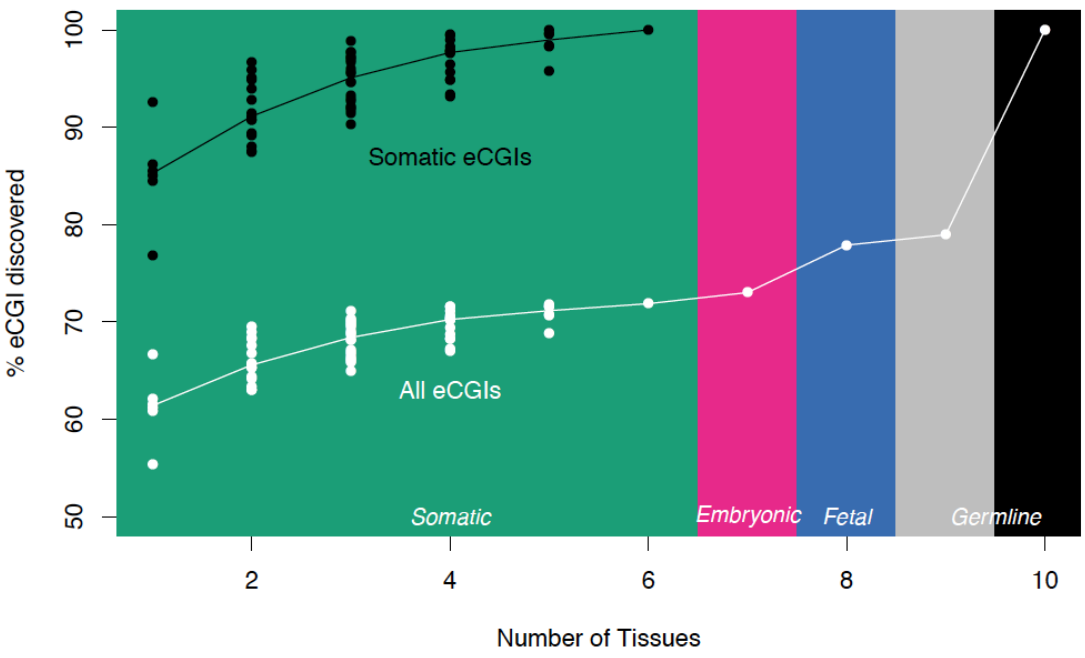

Figure S3. Autocorrelation values of CpG methylation values in chr 19 in B-cell at different distance windows. A. up to 50 kb. B. a zoom up to 2kb. The figures show that autocorrelation values further than 500 bp are relatively low ($r^2<0.4$). C. Number of eCGIs detected if all adjacent segments (within a given distance, x axis) are merged. D. Percentage of merged eCGIs that would overlap with cCGIs (i.e. non-novel eCGIs). Importantly, to get this estimates we merged all adjacent eCGIs, including methylated or CpG free regions in-between eCGIs, which is a conservative estimate.

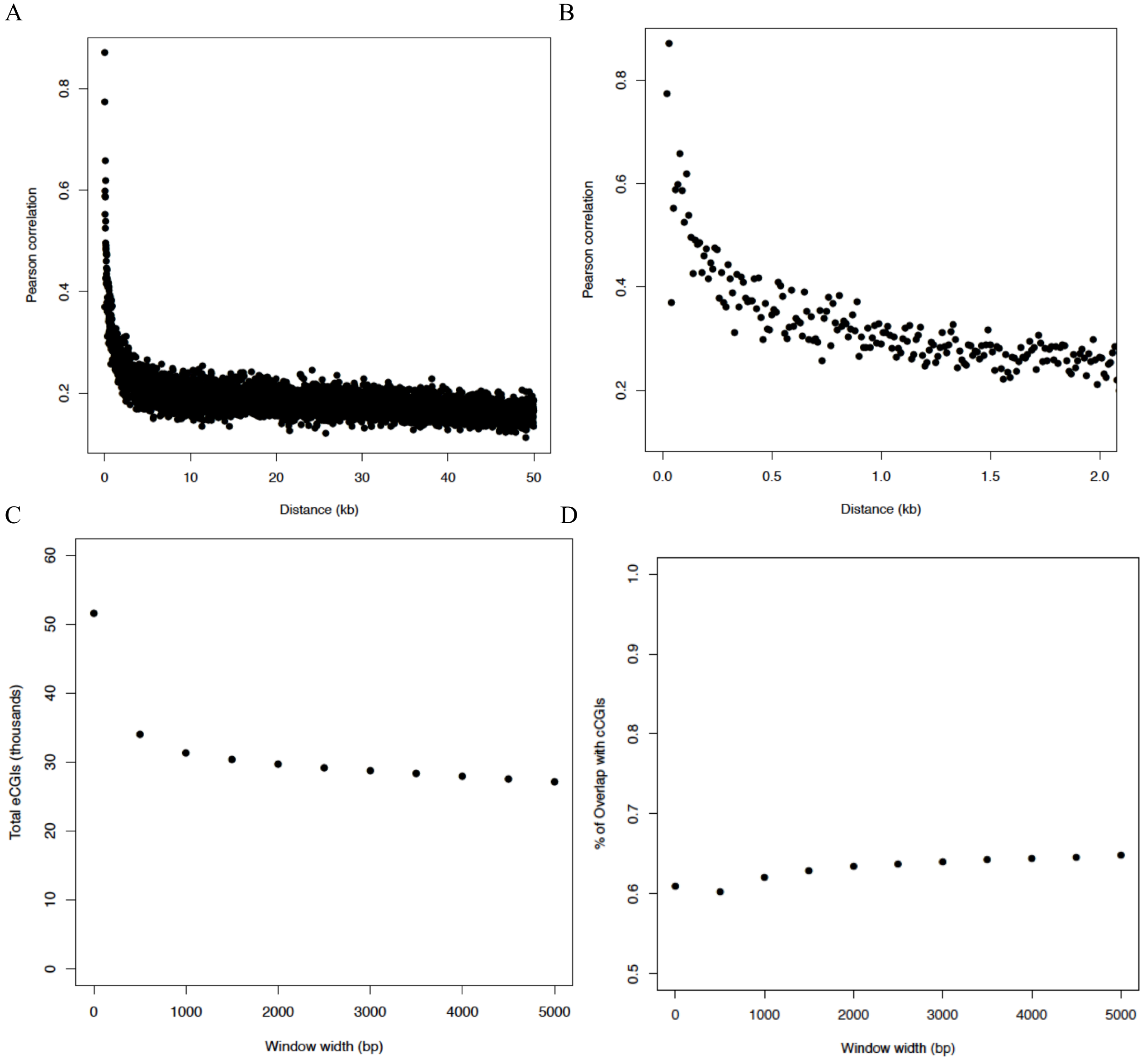

Figure S4. Examples of novel constitutive eCGIs in gene promoters. These include ubiquitously expressed genes such as (A) C1D (apoptosis-inducing), (B) COMMD1 (copper homeostasis). These eCGIs additionally overlap with active promoter chromatin marks and with TSS in CAGE data in B-cell.

A.

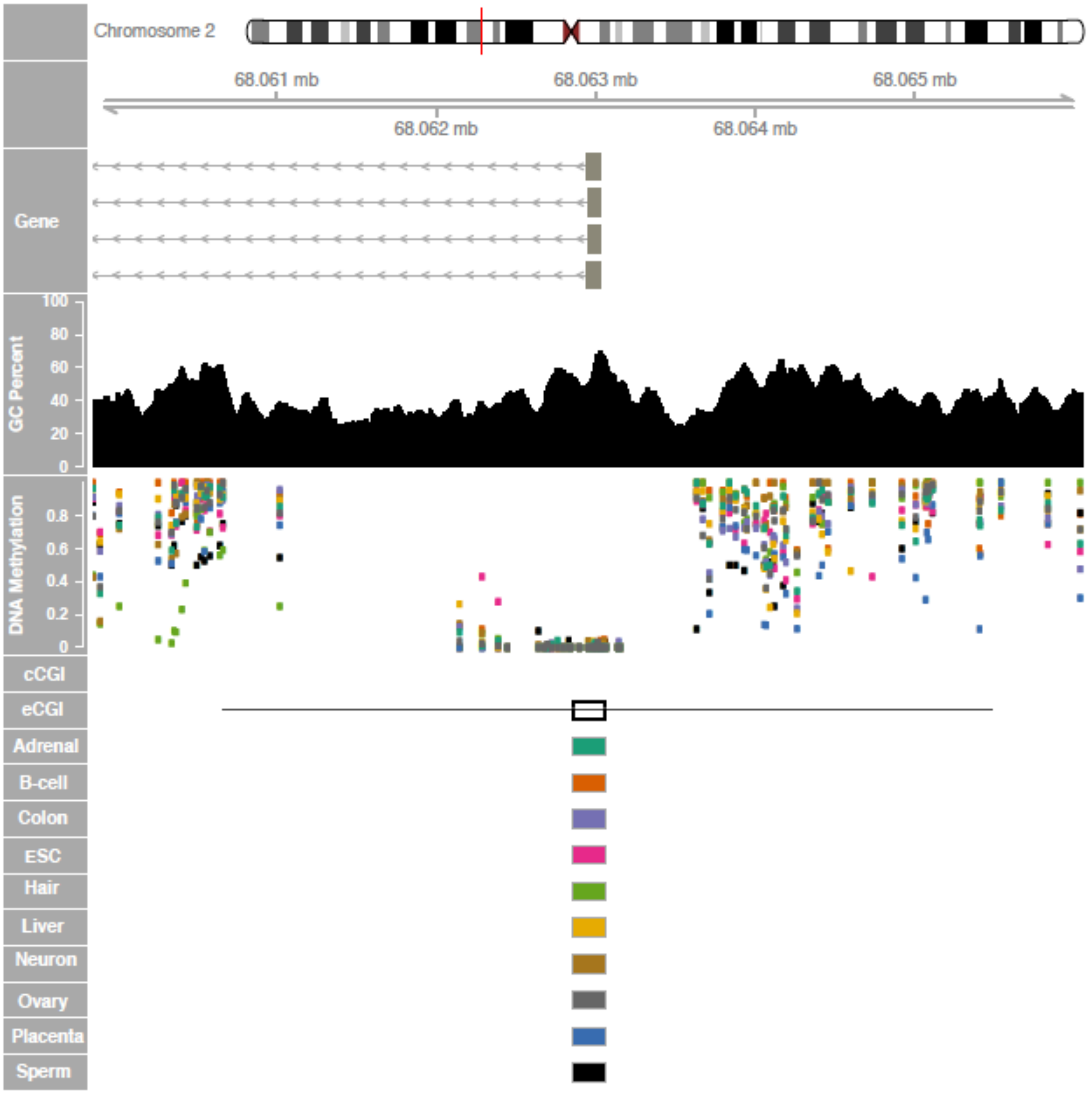

B.

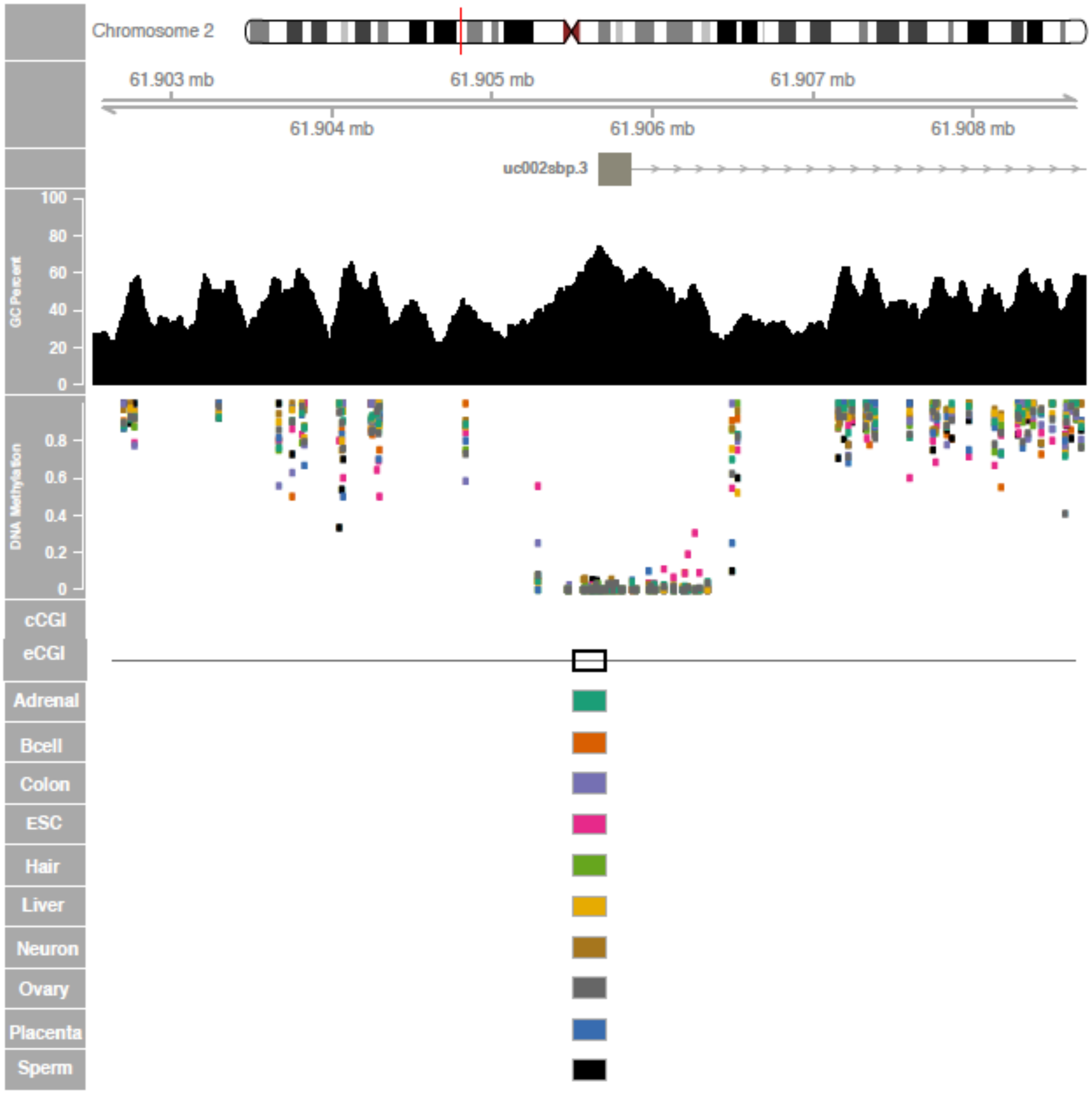
Chromosome 2
61.903 mb
61.905 mb
61.907 mb
61.904 mb
61.906 mb
61.908 mb
uc002sbp.3
GC Percent
100
80
60
40
20
0
DNA Methylation
0.8
0.6
0.4
0.2
0
cCGI
eCGI
Adrenal
Bcell
Colon
ESC
Hair
Liver
Neuron
Ovary
Placenta
Sperm

Figure S5. Sequence contents of computational (cCGI) and experimental CGI (eCGI) sets. "Novel eCGIs" represent the set of eCGIs that do not overlap with cCGIs. Constitutive and tissue-specific eCGIs represent eCGIs hypomethylated in 1 and 10 tissues out of 10, respectively). A. CGI length; B. CpG density (number of CpGs/CGI length); C. CpG O/E; D. GC content.

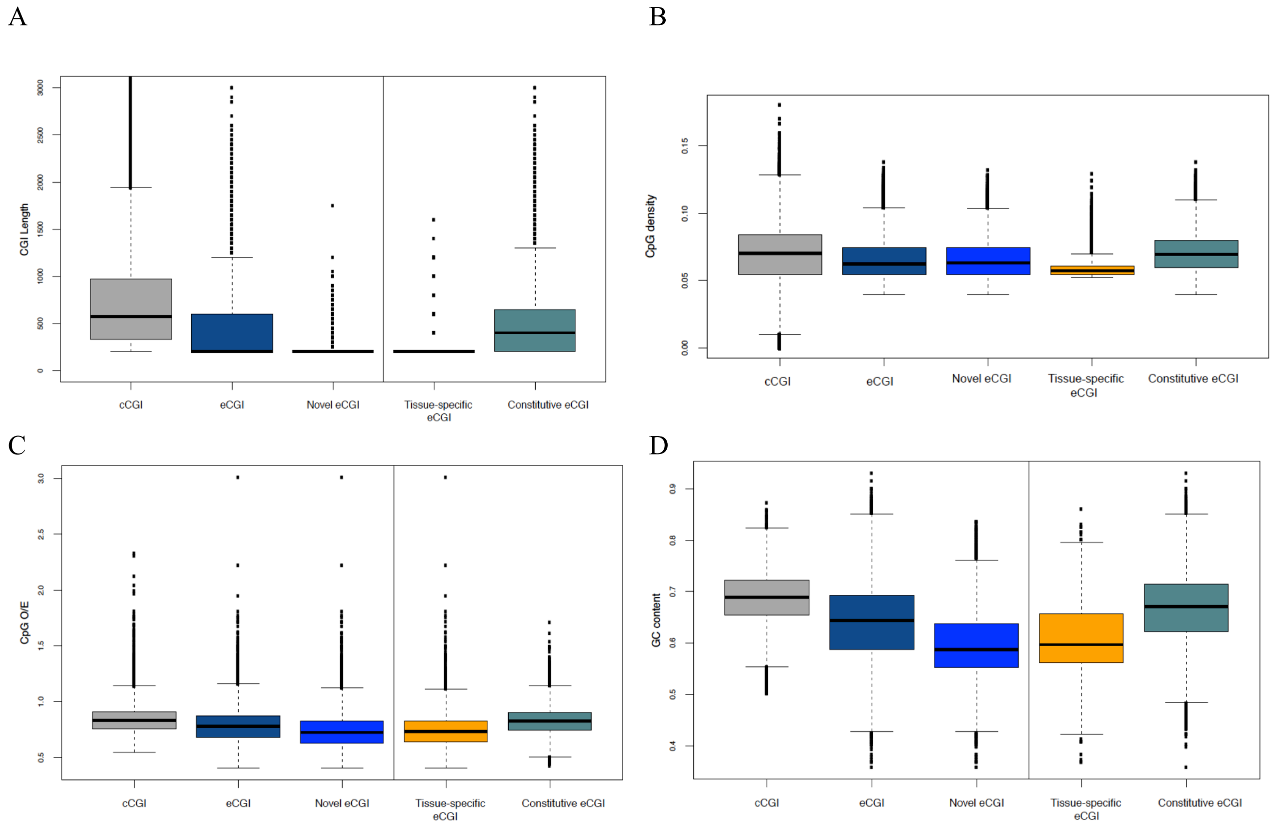

Figure S6. Repetitive elements (repeatmasker) at CGIs. Tissue-specific and constitutive eCGIs refer to eCGIs hypometylated in 1 and 10 tissues respectively.

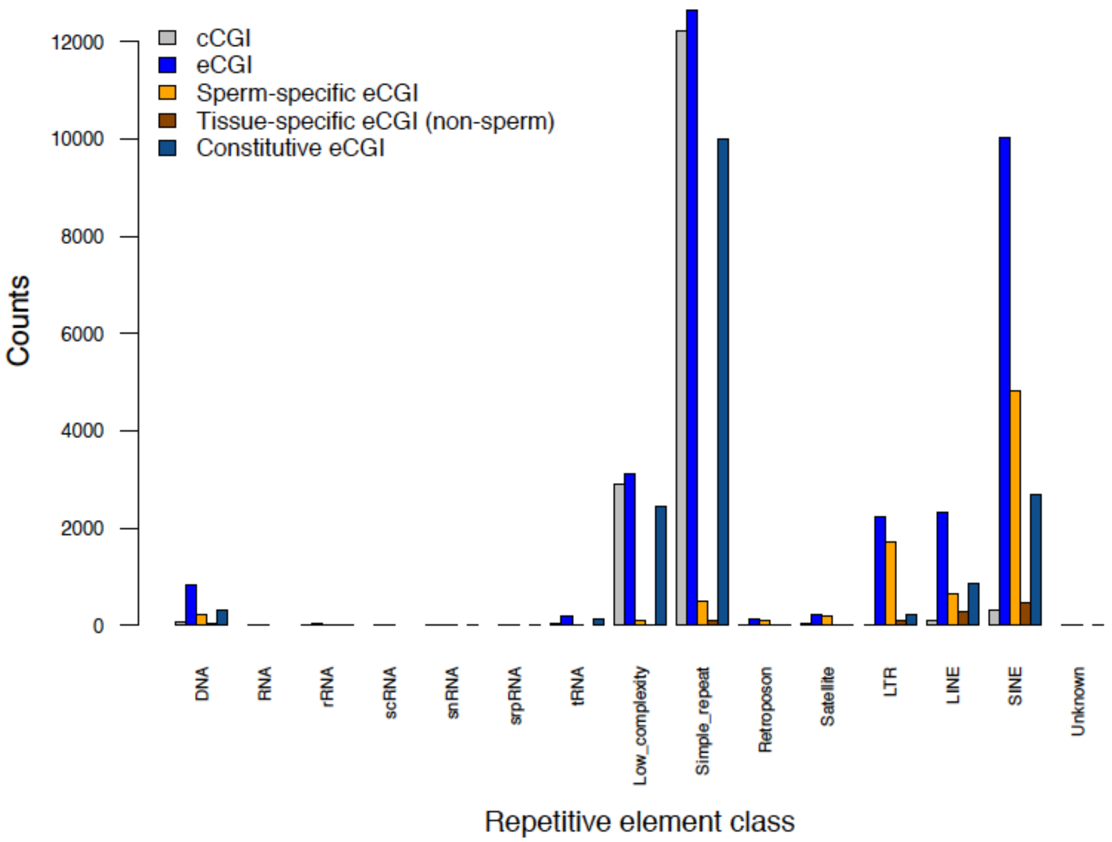

Figure S7. Hypomethylation breadth and number of TSS and TFs they harbor. A. Correlation between hypomethylation breadth and TSS breadth. TSS breadth indicates the number of FANTOM5 tissues in which a given CGI harbors at least one TSS (CAGE data). B. Correlation between hypomethylation breadth and the number of transcription factors that eCGIs overlap with.. C Histogram showing the distributions of eCGI and cCGI TSS breadths with a minimum 3 tags supporting each TSS per tissue. D Same as panel C but at least 5 tags are required to call a TSS in each tissue. For comparison, Figure 5A shows the plots for a minimum of 1 tag per TSS in each tissue.

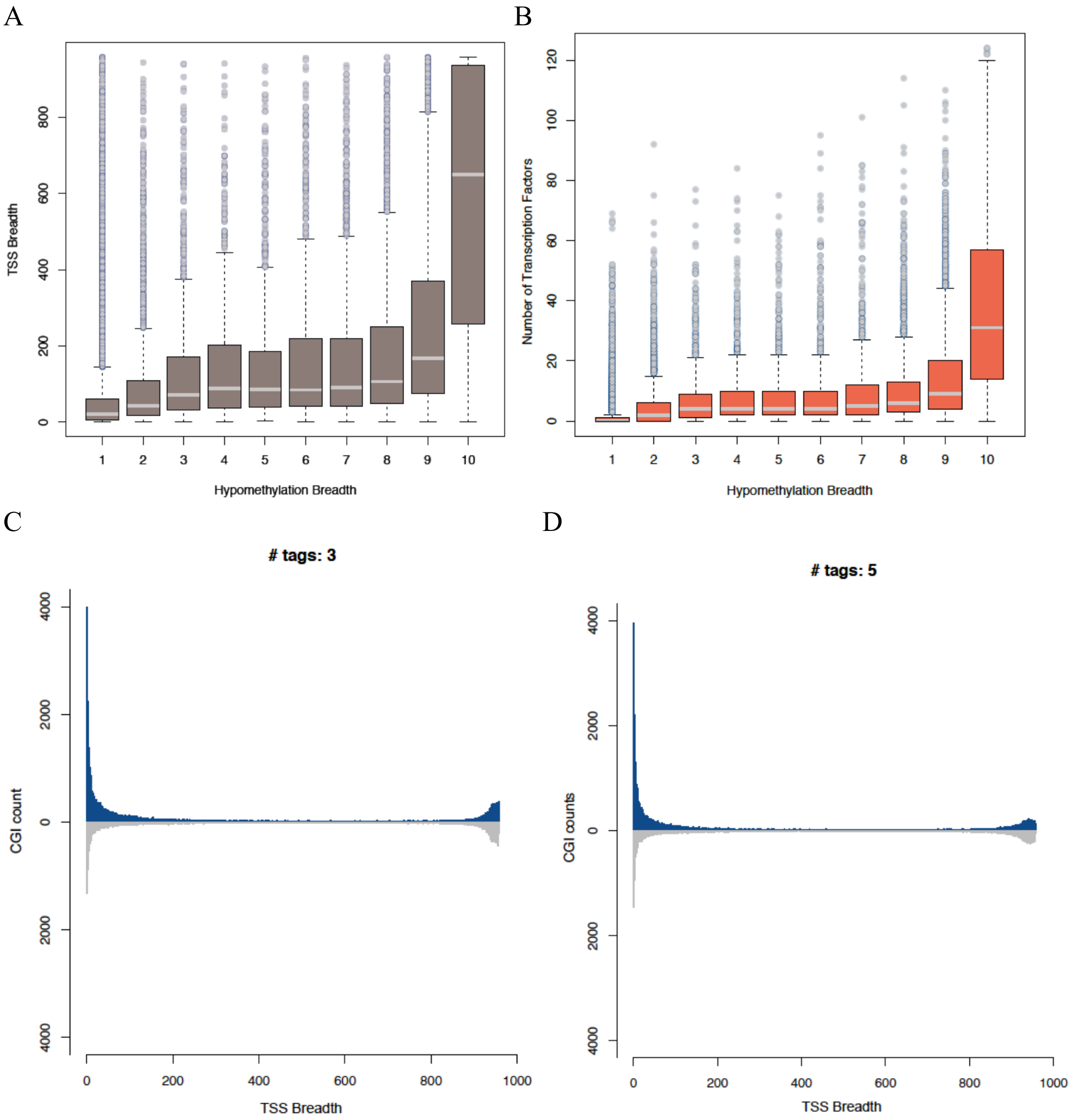

Figure S8. Correlation between promoter methylation and gene expression (BioGPS dataset) in B cell. Each dot corresponds to a gene. Promoter methylation values correspond to 200bp around TSS.

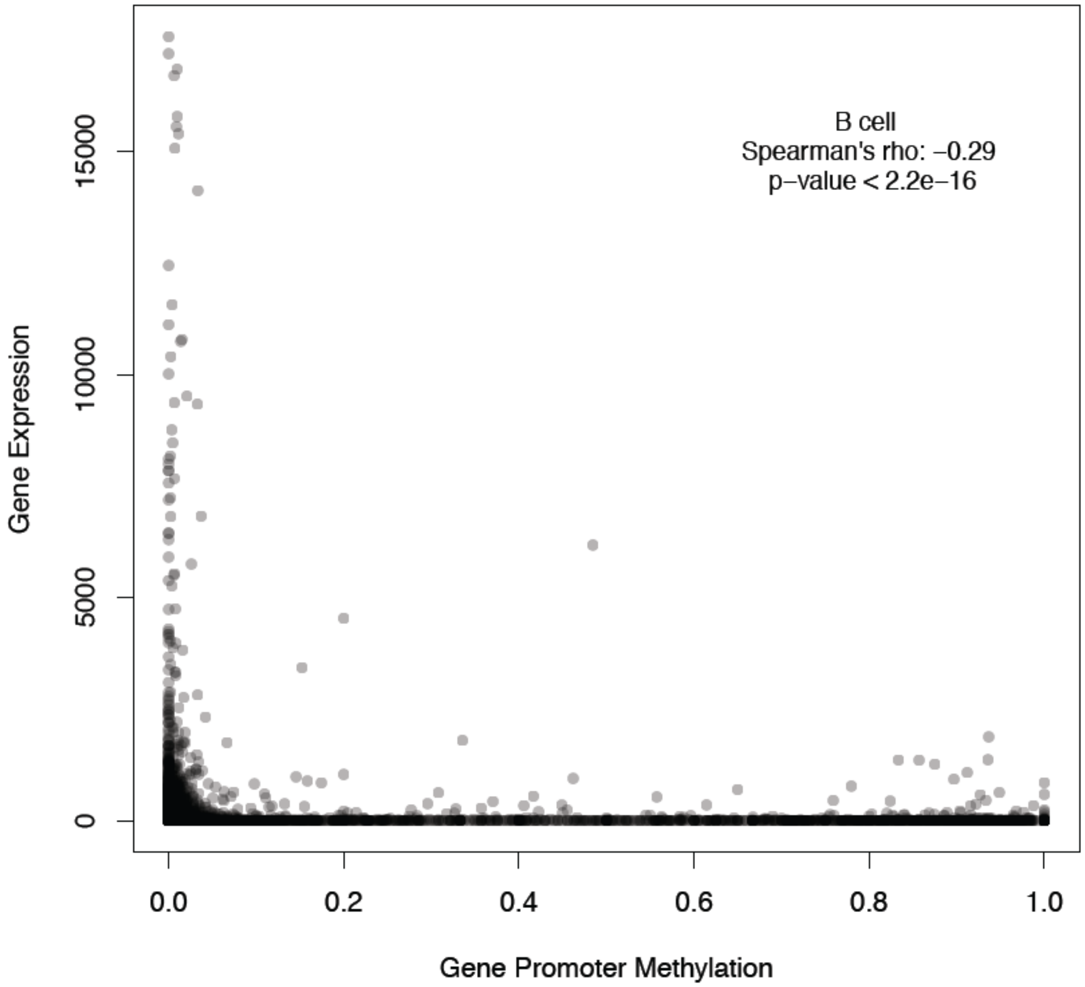

Figure S9. Expression breadth of tissue-specific eCGIs versus constitutive eCGIs. The expression breadth of each gene was computed as the number of tissues showing expression values above 0.25, 0.50 or 0.75 quantiles across all tissues. The upper figures show the genomic distribution of breadths according to those thresholds (from left to right: 0.25, 0.50 and 0.75 quantiles). The lower figures show the distribution of expression breadth between tissue-specific versus constitutive eCGIs at promoter genes according to those thresholds (from left to right: 0.25, 0.50 and 0.75 quantiles). Independently of the cutoff used, genes with tissue-specific eCGIs in their promoters show a more tissue-specific expression distribution than constitutive eCGIs. We defined tissue-specific promoter genes as those with at least one tissue-specific eCGI and no constitutive eCGIs in their promoters. Constitutive genes were defined as genes containing constitutive eCGIs and no tissue-specific eCGIs in the promoter region.

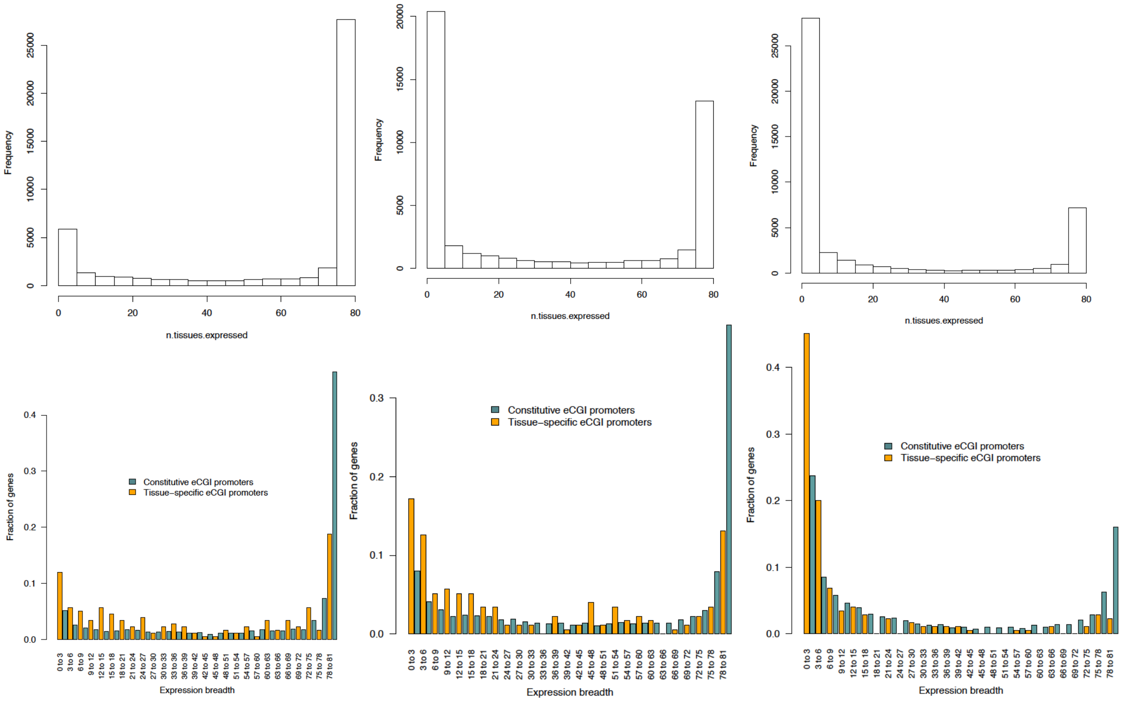

Figure S10. Fraction of non-validated (methylated) computational CGIs according to different gene biotypes.

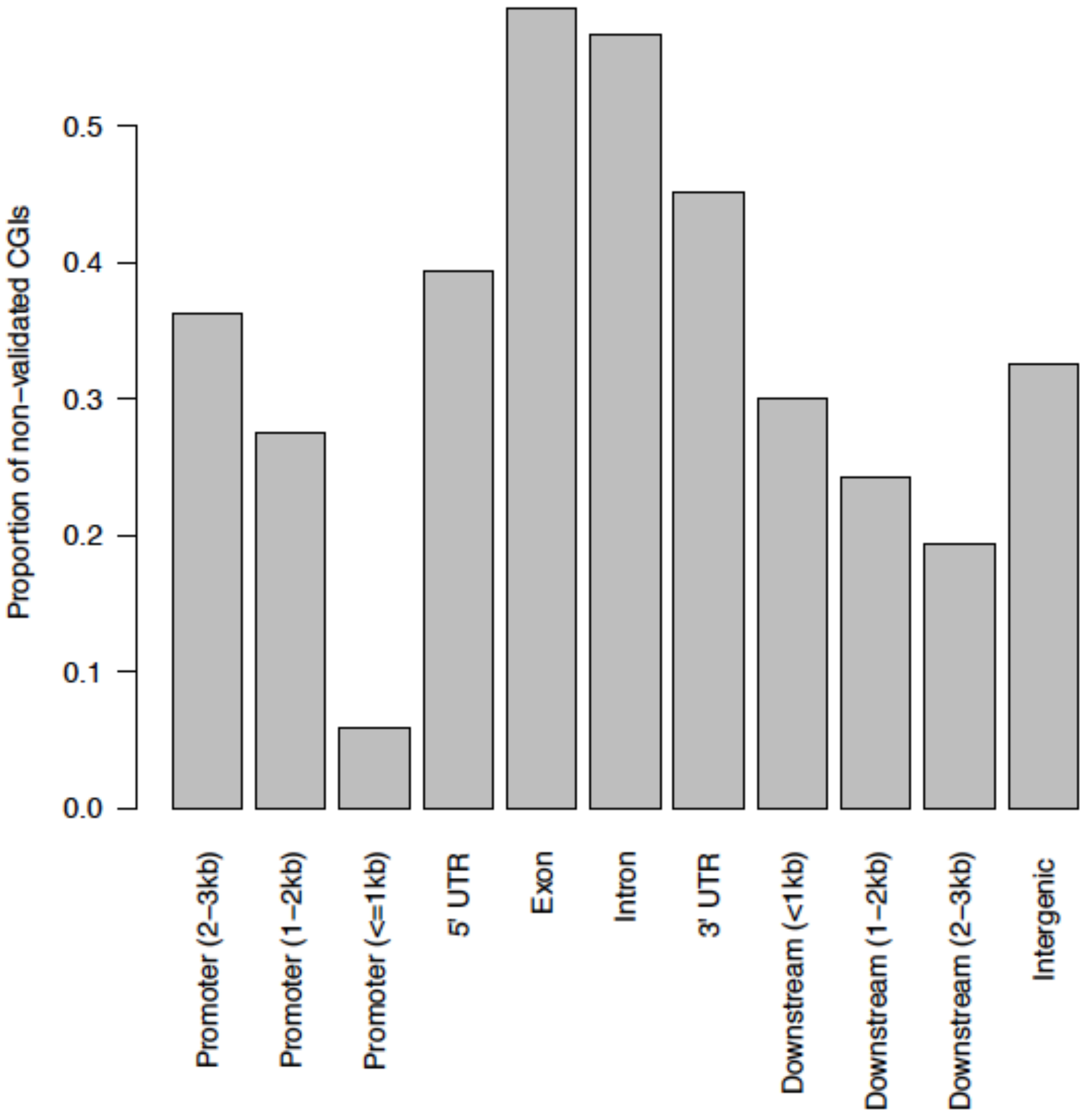

Figure S11. Number of tissues that show eCGI hypomethylation according to distance from nearest gene.

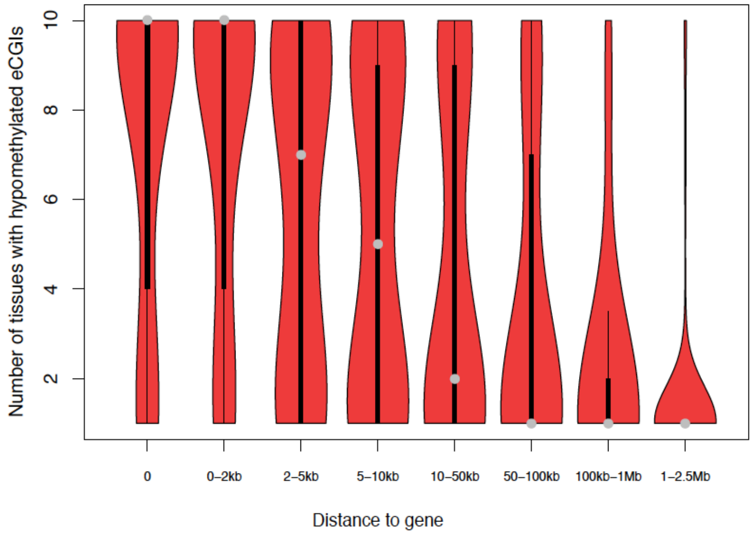

Figure S12. Comparison of sequence features of CGI sets. To compare with other experimental CGI sets (Long et al and Illingworth et al), we run our algorithm using different CpG density cutoffs. The density setting used in the manuscript for comparison to computational CpG islands is eCGI_10, which represents a density larger than 10 CpGs per 200 bp. We additionally studied other cutoff densities (15, 5 and 1, which we refer to as eCGI_15, eCGI_5, and eCGI_1). Figure panels:

A. Total MB of CGIs.

B Length distribution

C. CpG O/E

D. GC content

E. Fraction of CGI length showing overlap with transposable elements.

F. Venn diagram showing the overlap among CGI sets. Note that area is proportional to number of CGIs, not total MB (i.e. eCGI 5 is about half of Long et al dataset in total length, see Panel A). Illingworth et al.'s study in particular, captures the most CpG rich islands (most of them also predicted by computational methods, cCGI). On the other hand, Long et al. report 99 MBs of CGIs, which is considerably larger fraction of the genome that Illingworth et al dataset (25MB) or our CpG islands at minimum density of 10 CpGs per 200 bp (21 MB). However, setting our method to capture lower densities (>5 CpGs per 200bp, eCGI_5) only sums 53 MB of CGIs but contains the vast majority of Long et al annotations, providing a larger fraction of novel CGIs not detected by any previous study. These results suggest that in comparable settings our method improves CGI discovery.

A

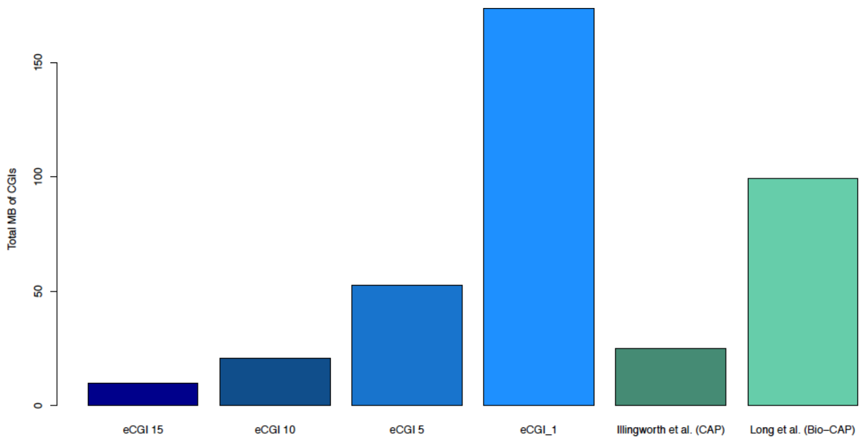

Total MB of CGIs
0
50
100
150
eCGI 15
eCGI 10
eCGI 5
eCGI_1
Illingworth et al. (CAP)
Long et al. (Bio–CAP)

B

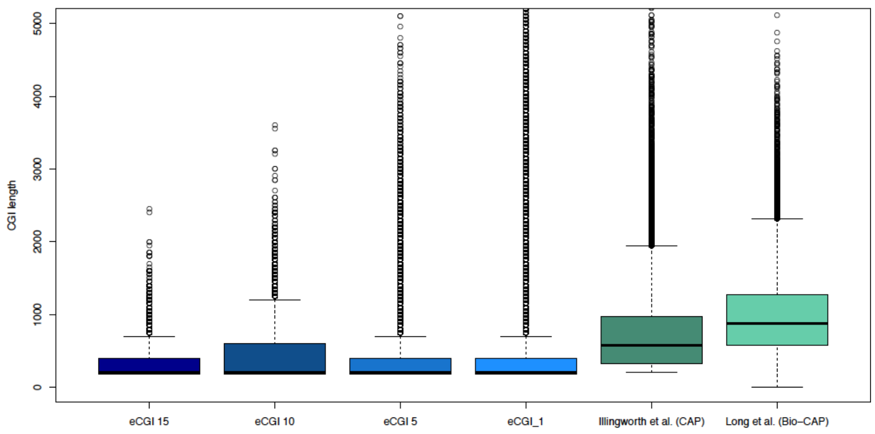

CGI length
0
1000
2000
3000
4000
5000
eCGI 15
eCGI 10
eCGI 5
eCGI_1
Illingworth et al. (CAP)
Long et al. (Bio–CAP)

C

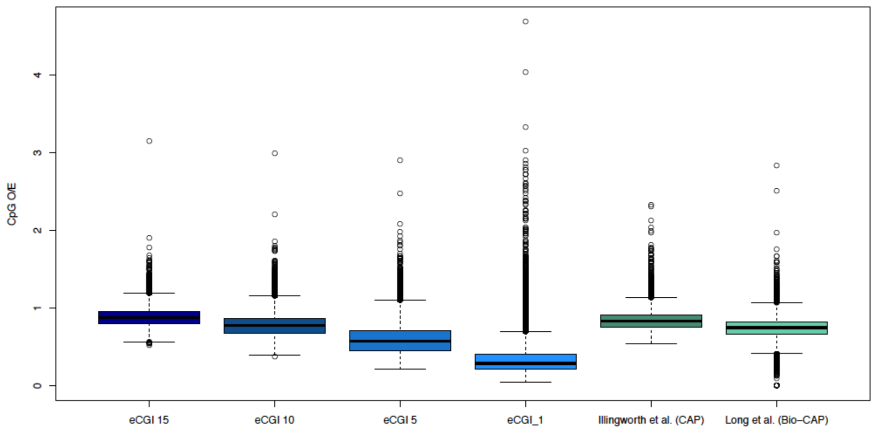

CpG O/E
0
1
2
3
4
eCGI 15
eCGI 10
eCGI 5
eCGI_1
Illingworth et al. (CAP)
Long et al. (Bio–CAP)

D

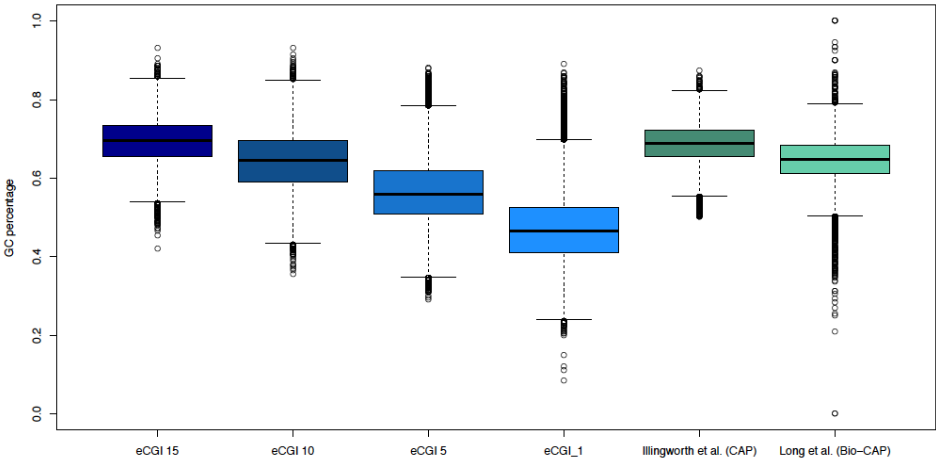

GC percentage
0.0
0.2
0.4
0.6
0.8
1.0
eCGI 15
eCGI 10
eCGI 5
eCGI_1
Illingworth et al. (CAP)
Long et al. (Bio–CAP)

E

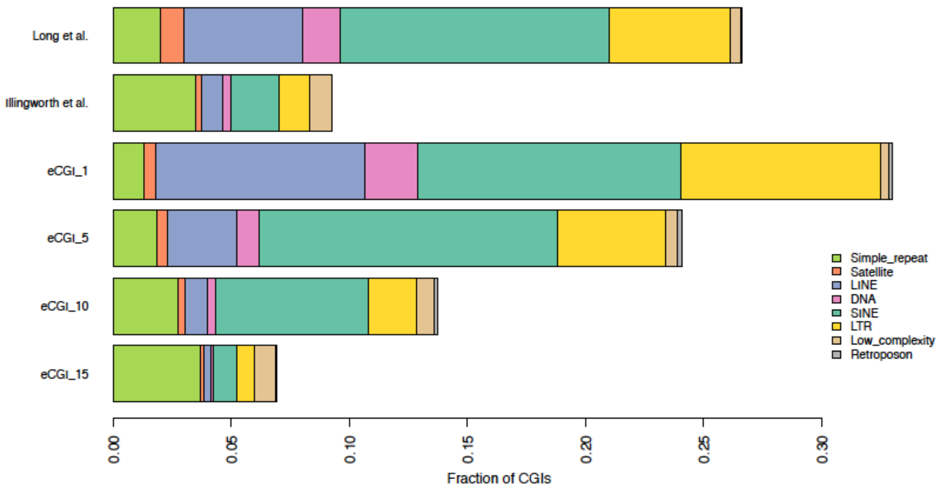

Long et al.
Illingworth et al.
eCGI_1
eCGI_5
eCGI_10
eCGI_15
Simple_repeat
Satellite
LINE
DNA
SINE
LTR
Low_complexity
Retroposon
0.00
0.05
0.10
0.15
0.20
0.25
0.30
Fraction of CGIs

F

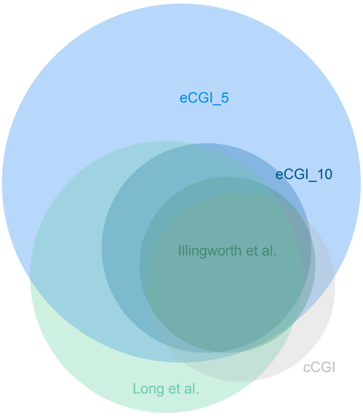

eCGI_5
eCGI_10
Illingworth et al.
cCGI
Long et al.

Figure S13. eCGI characteristics per tissue. A. Total number of eCGI, B. Length distribution of eCGI in bp C. Total length (cumulative) of eCGI in Mb. D. CpG observed/expected ratios.

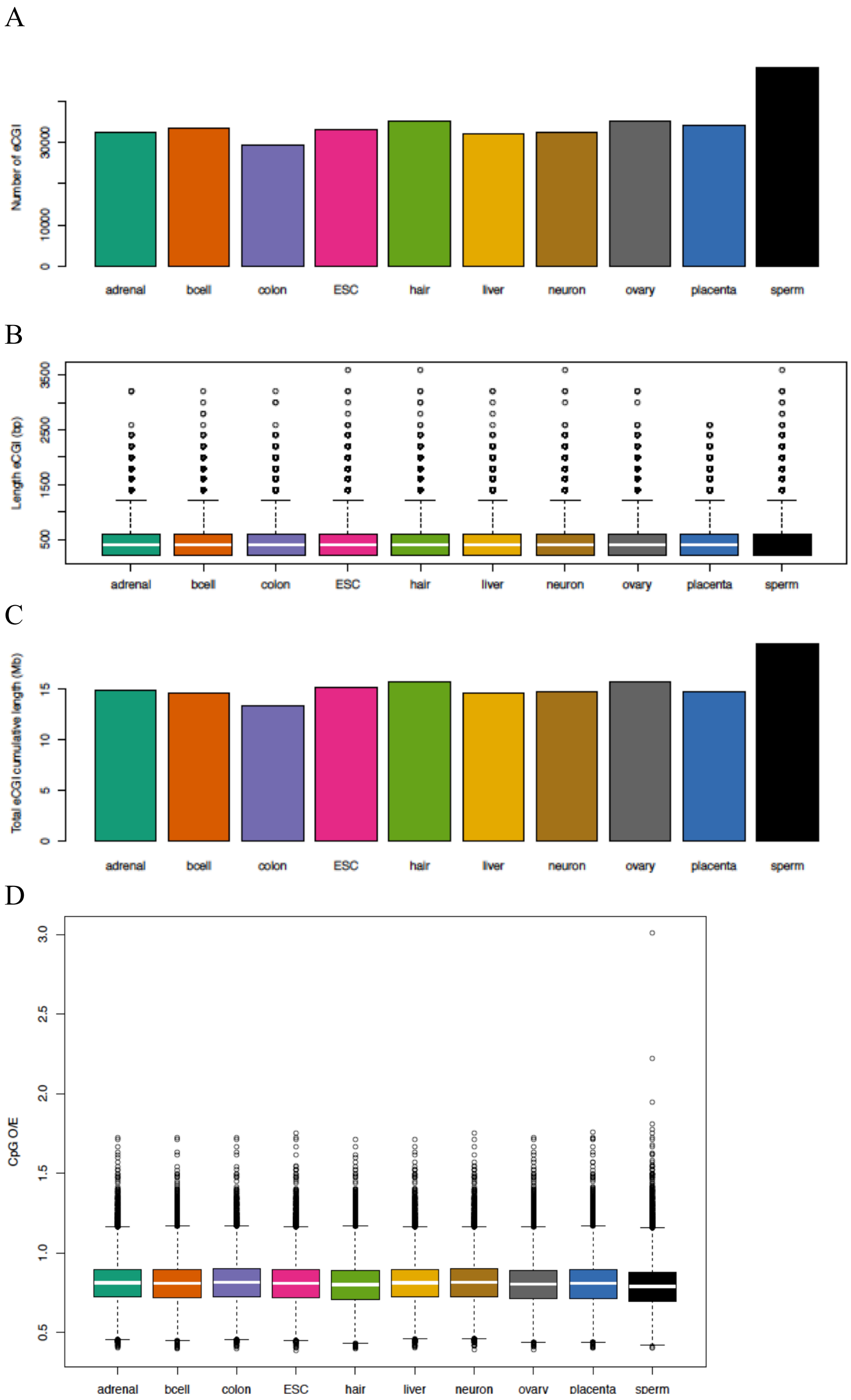

Figure S14. Tissue distribution of eCGIs defined by using different CpG density criteria. Topleft: > 1CpG per 200 window. Topright: > 5 CpG per 200 window. Bottomleft: > 10 CpGs per 200 per window. Bottomright: >15 CpGs per 200 window. As the minimum CpG number is set at higher thresholds, the composition of the CGI set changes to more a constitutive and less tissue-specific distribution.

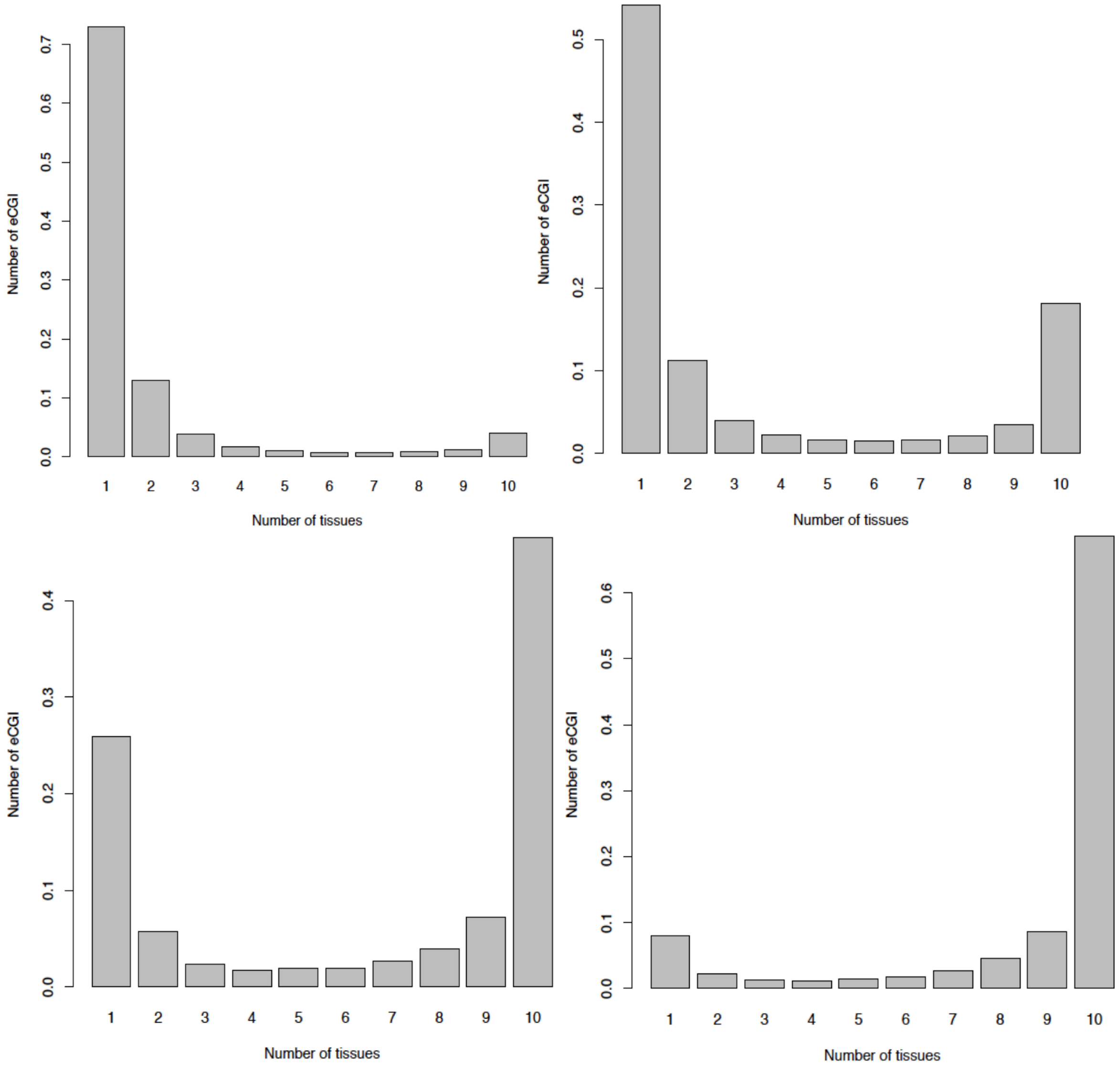

Supplementary Tables

Table S2. Enrichment for transcription factors at different CGI classes. For each CGI set, we counted the number of peaks observed for each transcription factor. We bootstrapped the same number of peaks randomly (n=10,000) and counted the number of random sets that showed values bigger or equal than the observed ones ($P<0.05$ shown).

CGI set	Transcription factor
Constitutive	ATF1 ATF3 BACH1 BRCA1 BRF1 CEBPD CREB1 ELK4 ESRRA GRp20 GTF2F1 HDAC6 HSF1 IRF1 IRF3 KDM5A POLR3G PPARGC1A RDBP RFX5 SAP30 SIX5 SREBP1 SRF STAT1 STAT2 TAF7 THAP1
B-cell specific	ATF2 BCL11A EBF1 FOXM1 IKZF1 IRF4 MEF2A MEF2C NFATC1 NFIC RUNX3 SPI1 STAT5A USF2
ESC specific	NANOG
Hair specific	ESR1 FOSL2 NR3C1 STAT3
Neuron specific	RCOR1
Placenta specific	SMC3
Ovary specific	HNF4A
Sperm specific	RAD21 SETDB1 ZNF143

Table S3. Correlation values (Spearman's rho) between the differential promoter methylation and gene expression of tissue pairs.

Source	*Dataset*	*tissue1*	*tissue2*	*rho*	*pvalue*
BioGPS	Affymetrix Human Genome U133A Array	adrenal	bcell	-0.11	4.51E-25
BioGPS	Affymetrix Human Genome U133A Array	adrenal	liver	-0.10	4.31E-20
BioGPS	Affymetrix Human Genome U133A Array	adrenal	ovary	-0.07	1.25E-10
BioGPS	Affymetrix Human Genome U133A Array	adrenal	placenta	-0.03	2.83E-03
BioGPS	Affymetrix Human Genome U133A Array	adrenal	neuron	-0.18	8.07E-63
BioGPS	Affymetrix Human Genome U133A Array	adrenal	colon	-0.07	9.01E-10
BioGPS	Affymetrix Human Genome U133A Array	bcell	liver	-0.08	6.28E-14
BioGPS	Affymetrix Human Genome U133A Array	bcell	ovary	-0.17	2.60E-51
BioGPS	Affymetrix Human Genome U133A Array	bcell	placenta	-0.10	2.60E-20
BioGPS	Affymetrix Human Genome U133A Array	bcell	neuron	-0.09	1.32E-15
BioGPS	Affymetrix Human Genome U133A Array	bcell	colon	0.00	7.31E-01
BioGPS	Affymetrix Human Genome U133A Array	liver	ovary	-0.11	1.76E-24
BioGPS	Affymetrix Human Genome U133A Array	liver	placenta	-0.04	8.08E-05
BioGPS	Affymetrix Human Genome U133A Array	liver	neuron	-0.17	3.90E-55
BioGPS	Affymetrix Human Genome U133A Array	liver	colon	-0.05	1.61E-06
BioGPS	Affymetrix Human Genome U133A Array	ovary	placenta	-0.03	1.67E-03
BioGPS	Affymetrix Human Genome U133A Array	ovary	neuron	-0.22	8.37E-90
BioGPS	Affymetrix Human Genome U133A Array	ovary	colon	-0.10	2.03E-18
BioGPS	Affymetrix Human Genome U133A Array	placenta	neuron	-0.14	1.65E-37
BioGPS	Affymetrix Human Genome U133A Array	placenta	colon	-0.03	4.62E-03
BioGPS	Affymetrix Human Genome U133A Array	neuron	colon	-0.12	2.39E-28
RNA-Seq Atlas	RNA-seq	colon	neuron	-0.23	1.39E-92
RNA-Seq Atlas	RNA-seq	colon	ovary	-0.01	5.45E-01
RNA-Seq Atlas	RNA-seq	colon	liver	-0.07	2.73E-09
RNA-Seq Atlas	RNA-seq	neuron	ovary	-0.1	6.51E-19
RNA-Seq Atlas	RNA-seq	neuron	liver	-0.23	8.03E-88
RNA-Seq Atlas	RNA-seq	ovary	liver	-0.22	9.25E-82

Table S4. Correlation between sequence content of promoter regions and methylation breadth with gene expression breadth. CpG O/E ratio and GC content were computed 3kb around TSS of each gene with eCGIs in their promoter region.

	**Correlation with gene expression breadth (BioGPS database)**	
	**Spearman's Rho**	**P-value**
**GC content**	-0.084	P= 1.8 e-12
**CpG O/E ratio**	0.081	P= 9.9 e-12
**Methylation Breadth**	0.17	P < 2.2e-16

Table S5. GO enrichments for tissue-specific eCGI promoter genes at each tissue. Some tissues (liver, adrenal gland, and colon) did not have genes exclusively with tissue-specific eCGIs at their promoters and were excluded from the analysis. First 5 results for enrichments at Biological Process (BP) and p-value < 0.05 are shown. In the case of neuron no significant results were found for BP, and CC (Celular Component) is shown instead. Genes with tissue-specific eCGIs in their promoters are enriched for tissue-specific GO functional categories (i.e. B-cells for immunity, ovaries for female gonad development, and sperm for spermatogenesis).

Gene classification	GOBPID	Pvalue	OddsRatio	Exp Count	Count	Size	Term
B-cell *Tissue-specific CGI Promoter Genes*							
	GO:0006955	1.85E-06	11.01	1.76	10	993	immune_response
	GO:0002697	1.86E-05	19.59	0.35	5	197	regulation_of_immune_effector_process
	GO:0042113	6.89E-05	22.43	0.23	4	130	B_cell_activation
	GO:0045321	1.04E-04	10.37	0.82	6	463	leukocyte_activation
	GO:0033622	1.61E-04	140.09	0.02	2	11	integrin_activation
ESC *Tissue-specific CGI Promoter Genes*							
	GO:0016338	5.02E-03	315.44	0.01	1	18	calcium-independent_cell-cell_adhesion
	GO:0070830	8.91E-03	172.76	0.01	1	32	tight_junction_assembly
	GO:2001238	1.17E-02	130.50	0.01	1	42	positive_regulation_of_extrinsic_apoptotic_signaling_pathway
	GO:0046330	1.22E-02	124.41	0.01	1	44	positive_regulation_of_JNK_cascade
	GO:0071260	1.33E-02	113.78	0.01	1	48	cellular_response_to_mechanical_stimulus
Hair follicle *Tissue-specific CGI Promoter Genes*							
	GO:0042636	1.40E-03	Inf	0.00	1	1	negative_regulation_of_hair_cycle
	GO:0002159	2.79E-03	766.36	0.00	1	2	desmosome_assembly
	GO:0007207	4.18E-03	383.14	0.00	1	3	phospholipase_C-activating_G-protein_coupled_acetylcholine_receptor_signaling_pathway
	GO:0090280	8.35E-03	153.21	0.01	1	6	positive_regulation_of_calcium_ion_import
	GO:0006814	9.65E-03	15.42	0.15	2	108	sodium_ion_transport

Neuron *Tissue-specific CGI Promoter Genes*							
	GO:0042734	1.33E-02	101.76	0.01	1	40	presynaptic_membrane
	GO:0045211	4.80E-02	27.13	0.05	1	146	postsynaptic_membrane
Ovary *Tissue-specific CGI Promoter Genes*							
	GO:0034635	1.67E-03	1341.88	0.00	1	2	glutathione_transport
	GO:2000195	1.67E-03	1341.88	0.00	1	2	negative_regulation_of_female_gonad_development
	GO:0007538	2.51E-03	670.88	0.00	1	3	primary_sex_determination
	GO:0031427	3.35E-03	447.21	0.00	1	4	response_to_methotrexate
	GO:0001553	4.18E-03	335.38	0.00	1	5	luteinization
Placenta *Tissue-specific CGI Promoter Genes*							
	GO:0046530	2.94E-03	28.27	0.08	2	38	photoreceptor_cell_differentiation
	GO:0035845	4.28E-03	487.32	0.00	1	2	photoreceptor_cell_outer_segment_organization
	GO:0051549	4.28E-03	487.32	0.00	1	2	positive_regulation_of_keratinocyte_migration
	GO:0071482	6.74E-03	18.14	0.12	2	58	cellular_response_to_light_stimulus
	GO:0007603	9.16E-03	15.38	0.15	2	68	phototransduction,_visible_light
Sperm *Tissue-specific CGI Promoter Genes*							
	GO:0007283	1.52E-15	7.34	5.36	31	291	spermatogenesis
	GO:0044703	7.77E-13	4.59	10.58	39	574	multi-organism_reproductive_process
	GO:0032504	9.86E-13	4.87	9.14	36	496	multicellular_organism_reproduction
	GO:0043046	1.01E-09	64.39	0.24	7	13	DNA_methylation_involved_in_gamete_generation
	GO:0034587	2.91E-09	109.83	0.17	6	9	piRNA_metabolic_process

Table S6. **Enrichment values of different chromatin states at novel intergenic eCGIs.** Results for B-lymphoblastoid cell are shown.

Chromatin states	***Observed***	**Expected Mean**	**Expected SD**	**Enrichment**	**P-value**
1_Active_Promoter	1702	60.18	7.53	28.28	<0.001
10_Txn_Elongation	53	398.47	19.31	0.13	1
11_Weak_Txn	302	760.88	25.62	0.40	1
12_Repressed	888	231.73	14.73	3.83	<0.001
13_Heterochrom/lo	1267	4758.45	34.83	0.27	1
14_Repetitive/CNV	20	15.20	4.06	1.32	0.105
15_Repetitive/CNV	9	9.39	3.22	0.96	0.457
2_Weak_Promoter	830	65.91	8.21	12.59	<0.001
3_Poised_Promoter	551	14.14	3.73	38.97	<0.001
4_Strong_Enhancer	152	73.26	8.33	2.07	<0.001
5_Strong_Enhancer	35	77.60	8.61	0.45	1
6_Weak_Enhancer	489	100.29	9.51	4.88	<0.001
7_Weak_Enhancer	83	204.07	14.58	0.41	1
8_Insulator	153	53.98	7.24	2.83	<0.001
9_Txn_Transition	21	57.02	7.55	0.37	1

Table S7. Comparative table for studies of experimentally defined CGIs. In blue, the density setting used in this study for comparison to computational CpG islands. To compare with other experimental CGI sets (Long et al and Illingworth et al), we ran our algorithm using different CpG density cutoffs (1, 5 and 15). See Figure S12 for additional comparison of sequence features. The CGIs from liver (n=35,325) and testes (n=40,697) from Long et al. were reduced to non-overlapping CGIs as done for our CGIs from ten tissues.

	Illingworth et al.	***Long et al.***	***This study***			
Minimum CpG density (# CpGs per 200bp)	Unknown	Unknown	*>1*	*>5*	*>10*	*>15*
Method	CAP	Bio-CAP	Bisulfite sequencing			
Total_number	24,778	50,450	494,095	125,086	51,572	29,356
Total MB	25	99.24	173.4	52.6	20.9	9.7
Median CGI length (bp)	891	1583	200	200	200	200
Mean CGI length (bp)	1011	1967	351	420.9	405	331.4
Mean CpG O/E	0.84	0.74	0.34	0.59	0.78	0.89
Mean GC content	0.69	0.65	0.47	0.57	0.64	0.69
TE overlap (fraction of total MB)	0.09	0.27	0.33	0.24	0.14	0.07

Table S8. Functional fraction of CGIs from different catalogues. The table shows the average fraction of the CGI length that overlaps with regulatory elements identified by ChIP-seq experiments: transcription factor binding regions (91 tissues), promoter and enhancers / insulators in lung fibroblast cells (cell-type not included in CGI discovery in any of the studies). For each CGI set, only CGIs showing overlap with ChIP-seq peaks are included in this comparison.

			**Fraction of CGI length with functional elements**		
**CGI set**	**Method**	**#tissues in CGI discovery**	**Transcription Factors**	**Promoters**	**Enhancer and insulators**
This study	Bisulphite sequencing	10	0.96	0.68	0.39
Illingworth et al. (24)	CXXC affinity purification (CAP)	3	0.94	0.51	0.22
Long et al. (22)	CXXC affinity purification (Bio-CAP)	2	0.67	0.24	0.15
UCSC	Computational prediction	0	0.93	0.50	0.20

Table S9. Samples used in this study.

**Tissue**	**REFERENCE**	**GEO ID**	***Experiment Accession***
colon	[5]	GSE46644	**SRX332737**
liver	[6]	GSE46698	**SRX275730**
placenta	[6]	GSE46698	**SRX275731**
ESC	[7]	GSE19418	**SRX015765**<br>**SRX015764**<br>**SRX015763**
Hair folicle	[9]	GSE44806	**SRX248434**
sperm	[10]	GSE30340	**SRX081760**<br>**SRX081759**<br>**SRX081761**<br>**SRX081762**
bcell	[11]	GSE31971	**SRX096520**<br>**SRX096517**
neuron	[12]	GSE47966	**SRX314938**
ovary	[13]	GSE16256	**SRX263879**<br>**SRX190152**
adrenal	[13]	GSE16256	**SRX190153**<br>**SRX263881**<br>**SRX263880**

Table S10. Number and length of reads analyzed at each step of quality control (QC) and mapping. Quality Control steps: Adaptive quality trimming and adapter trimming was performed using Trim Galore default settings (minimum read length of 20 and Phred score > 20). For paired end libraries "—trim1" option was applied to trim an additional base from the 3' ends to avoid overlapping of long reads. After this step, Bcell single end library and ESC did not pass the FastQC criteria and instead, specific adapter removal was performed with Cutadapt with those two samples. After these quality control steps, all samples passed the FastQC criteria. Mapping was performed with Bismark (using Bowtie) with the seed mismatch parameter "-n" set to 1 with other parameters set to default (-e 70). Reads with same orientation, start and end (probably originated from PCR amplification) were removed using Bismark de-duplication scripts.

	**GEO reads**	**After QC**	**Uniquely mapped**	**After de-duplication**	**Mean read length**
adrenal	2537943924	2477771404	2133851136	2027071816	100
bcell	679729554	550434598	389859540	317677499	93
colon	549135883	523383093	398373840	394825100	99
ESC	626379188	595922676	407343825	403508587	61
hair	457916830	448917975	354464940	335297335	100
liver	944657425	937852985	767863887	752035169	100
neuron	1474525499	1191479704	859358903	829920996	101
ovary	2792403509	2782246379	2389615185	2323563931	100
placenta	447848405	445467214	332628385	328007724	101
Sperm	749372383	772818193	427439483	416479647	93

Table S11. Statistics on mapping and methylation calling. Coverage indicates the fraction of genomic CpGs covered at each tissue. Depth indicates the number of reads covering each CpG position**.**

Tissues	Coverage 1x	Coverage 5x	Mean Depth >1x	Mean Depth >5x	Median Depth >1x	Median Depth >5x	Methylation
bcell	0.95	0.81	12.25	13.6	11.00	12.00	0.77
ESC	0.94	0.83	25.26	28.0	17.00	19.00	0.74
hair	0.94	0.88	26.54	28.4	24.00	26.00	0.73
neuron	0.93	0.90	25.93	26.7	23.00	24.00	0.82
placenta	0.93	0.84	29.44	32.4	24.00	26.00	0.62
colon	0.93	0.86	40.47	43.9	34.00	37.00	0.72
liver	0.97	0.95	60.01	61.3	56.00	57.00	0.74
adrenal	0.94	0.93	53.55	54.2	50.00	50.00	0.75
ovary	0.94	0.93	61.98	62.7	59.00	60.00	0.74
sperm	0.95	0.87	15.69	16.8	14.00	15.00	0.73

Table S12. eCGI discovery according to different settings. Results shown are from B-cell sample. The final setting used in the study is setting 1. The minimum percentage of hypomethylated CpGs per window was set originally to 80% (setting1), and when relaxed to 65% (settings 2 and 3) the number of eCGIs increased only in around 3.5% and the length distribution of eCGIs remained similar. Modest effects were also observed when the hypomethylation threshold (0.2 in settings 1 and 2) was set to 0.3 (setting 3).

	**eCGI criteria**			
	**# CpGs window**	**Window size (bp)**	**Hypomethylation criteria**	**% of hypomethylated CpGs per window**
Setting1	10	200	0.2	80
Setting2	10	200	0.2	65
Setting3	10	200	0.3	65

		**Length distribution eCGIs in B-cell**					
	**number eCGIs in B-cell**	**Min.**	**1stQu.**	**Median**	**Mean**	**3rdQu.**	**Max.**
Setting1	33368	201	201	401	440	601	3201
Setting2	34521	201	201	401	446	601	3201
Setting3	35481	201	201	401	447	601	3201

Table S13. **e**CGI discovery and functional overlap according to different CpG density settings. Tissue-specific category includes tissue-specific eCGIs from all tissues (including B-cell-specific eCGIs). The final setting used in the study is shown in blue. Functional fraction was computed as the fraction of eCGIs overlapping with active chromatin states in B-lymphoblastoid (promoter, enhancer, transcription, or insulator-related regions). In contrast, eCGIs overlapping repressed, heterochromatin, or CNV repetitive domains were considered "non-functional". Setting the CpG density at lower bounds (i.e. >1 CpG per 200 bp, 10x less than the final setting) allowed the discovery of 9.6 times more eCGIs and 8 times more MB of genomic eCGIs. In addition, this eCGI set is composed by 73% of tissue-specific eCGIs, and only 4% of classical constitutive eCGIs. The percentage of B-cell specific eCGIs that overlap with active histone marks is remarkably high even at lower densities (>=65%), and the fraction of "validated" constitutive eCGIs is always high (>=87%). Therefore, even if we considered the conservative set of >10CpG density to analyze further in this study, these results suggests that CGIs identified using our algorithm at more relaxed CpG density criteria are likely to be functionally relevant as well.

	Number of mapped CpGs per 200bp window			
	>1	***>5***	***>10***	***>15***
Total_number	494,095	125,086	51,572	29,356
Total MB	173.40	52.65	20.89	9.73
Fraction of constitutive eCGIs	0.04	0.18	0.47	0.69
Fraction of tissue-specific eCGIS	0.73	0.54	0.26	0.08
Fraction in cCGI (non-novel)	0.05	0.21	0.61	0.92
Minimum number if merge adjacent eCGIs within 5kb	143,383	60,366	27,134	16,676
Number of Bcell-specific eCGIs	24010	3986	298	22
Fraction of functional ALL	0.30	0.49	0.64	0.76
Fraction of functional constitutive	0.87	0.88	0.90	0.91
Fraction of functional tissue-specific	0.22	0.33	0.31	0.34
Fraction of functional B-cell-specific	0.65	0.78	0.81	0.91
